# The Agpat4/LPA axis in colorectal cancer cells regulates antitumor responses via p38/p65 signaling in macrophages

**DOI:** 10.1038/s41392-020-0117-y

**Published:** 2020-03-27

**Authors:** Dapeng Zhang, Rongchen Shi, Wei Xiang, Xia Kang, Bo Tang, Chuan Li, Linfeng Gao, Xuan Zhang, Lili Zhang, Rongyang Dai, Hongming Miao

**Affiliations:** 1 Department of Biochemistry and Molecular Biology, Southwest Medical University, Luzhou, 646000 China; 2 Department of Biochemistry and Molecular Biology, Third Military Medical University (Army Medical University), Chongqing, 400038 China; 3 Department of General Surgery, Southwest Hospital, Third Military Medical University (Army Medical University), Chongqing, 400038 China; 4 Department of Oncology, Southwest Hospital, Third Military Medical University (Army Medical University), Chongqing, 400038 China; 5 Department of Military Psychology, School of Psychology, Third Military Medical University (Army Medical University), Chongqing, 400038 China

**Keywords:** Cancer microenvironment, Tumour immunology, Cancer metabolism

## Abstract

Lipid metabolic reprogramming plays an essential role in regulating the progression of colorectal cancer (CRC). However, the effect of lysophosphatidic acid (LPA) metabolism on CRC development is incompletely characterized. Here, we compared the mRNA levels of human CRC tissues to those of paracarcinoma tissues and focused on the notably enriched LPA metabolic pathways. We identified and verified that 1-acylglycerol-3-phosphate O-acyltransferase 4 (Agpat4) was aberrantly expressed in CRC tissues and predicted poor survival in CRC patients. Manipulating Agpat4 expression in CRC cells did not affect the growth or migration of CRC cells in vitro, whereas Agpat4 silencing suppressed CRC cell growth in subcutaneous and peritoneal xenograft models. Mechanistically, Agpat4 silencing-induced LPA release from CRC cells and polarized macrophages to an M1-like phenotype through LPA receptors 1 and 3. This M1 activation, characterized by elevated p38/p65 signaling and increased proinflammatory cytokines, promoted the infiltration and activation of CD4^+^ and CD8^+^ T cells in the tumor microenvironment. Modulation of the Agpat4/LPA/p38/p65 axis regulated macrophage polarization, T-cell activity and CRC progression. Notably, combined therapy with LPA and regular chemotherapy drugs synergistically suppressed CRC development. Taken together, our results showed that the Agpat4/LPA axis in CRC cells regulated p38/p65 signaling-dependent macrophage polarization, T-cell activation, and CRC progression. The Agpat4/LPA/p38/p65 axis might represent a potential target for therapy in the clinic.

## Introduction

Colorectal cancer (CRC) is a serious disease that endangers human health. Among all cancers, CRC ranks third in morbidity and second in cancer-associated deaths.^1^ It is urgent to decipher the pathogenesis of CRC and develop corresponding strategies for the management of this disease.

Lipid metabolic reprogramming in the tumor microenvironment (TME) is essential for the development of solid tumors.^2–5^ The TME is composed of cell components (tumor cells, immune cells, endothelial cells, fibroblasts, etc.) and molecular components (cytokines, metabolites, etc.) and is developed under special conditions (hypoxia, nutrition deficiency, etc.).^6–8^ All cells in the TME need to actively or passively adjust their metabolism to adapt to and survive in this special environment. Recently, lipid metabolic reprogramming in cancer cells and cancer-associated immune cells has been intensively investigated.^3,5,9–11^

Lipid metabolic reprogramming can directly regulate the biological activities of cancer cells.^3,9,12,13^ In our previous studies, we found that neutral fat was deposited in human CRC cells due to deficiency in α/β-hydrolase domain-containing 5 (ABHD5), a coactivator involved in the lipolysis of triglycerides into diglycerides and free fatty acids.^14^ We demonstrated that ABHD5 deficiency switched the catabolism of fatty acids to glycolysis of glucose and promoted invasive activity in CRC cells.^14^ Suman K. Das et al. noted that functional lipolysis of triglycerides contributed to the pathogenesis of cancer-associated cachexia, and pharmacological inhibition of metabolic lipases prevented this disorder.^2^ In addition, phospholipid metabolism was also closely associated with the biological activity of cancer cells.^15–17^

Lipid metabolic reprogramming also occurs in tumor-associated immune cells and regulates the immune status of the TME.^5,10,18,19^ It was reported that fatty acid catabolism enhanced CD8^+^ T-cell efficacy in melanoma immunotherapy.^5^ Yang et al. reported that cholesterol was required for the antitumor activity of CD8^+^ T cells.^4^ Optimal antitumor responses require the integration of innate and adaptive immunity.^20^ Macrophages, the most abundant innate immune cells in the TME, are roughly classified into M1 and M2 phenotypes.^21^ M1-like macrophages, characterized by the cell surface markers CD45, F4/80 and CD11c as well as increased expression of the proinflammatory cytokines IL-1β, IL-6, and TNFα, exert antitumor functions by stimulating T-cell activation, which is characterized by increased production of IFN-γ.^19,20,21^ In contrast, M2-like macrophages, characterized by the protein markers CD45, F4/80, and CD206 as well as increased expression of the anti-inflammatory cytokines IL-10 and Arg-1, exhibit protumor functions by suppressing T-cell activity.^19,20,21^ We revealed that monoglyceride lipase deficiency resulted in lipid overload in tumor-associated macrophages and regulated cannabinoid receptor 2-dependent macrophage polarization, T-cell activation and CRC progression.^19^

Lipids consist of triglycerides, cholesterol, and phospholipids. Recent studies have mainly focused on the roles of triglyceride and cholesterol metabolism in cancer cells or immune cells.^9,22^ However, how phospholipid metabolism is reprogrammed in CRC cells and subsequently how the immune TME is regulated are still incompletely elucidated. In the present study, we screened lipid metabolic pathways in CRC tissues and identified the function of lysophosphatidic acid acyltransferase δ, also known as 1-acylglycerol-3-phosphate O-acyltransferase 4 (AGPAT4),^23^ in regulating the immune TME and CRC progression, as well as the mechanisms by which this regulation is accomplished.

## Results

### Aberrant expression of AGPAT4 in human CRC tissues

**Fig. 1:**
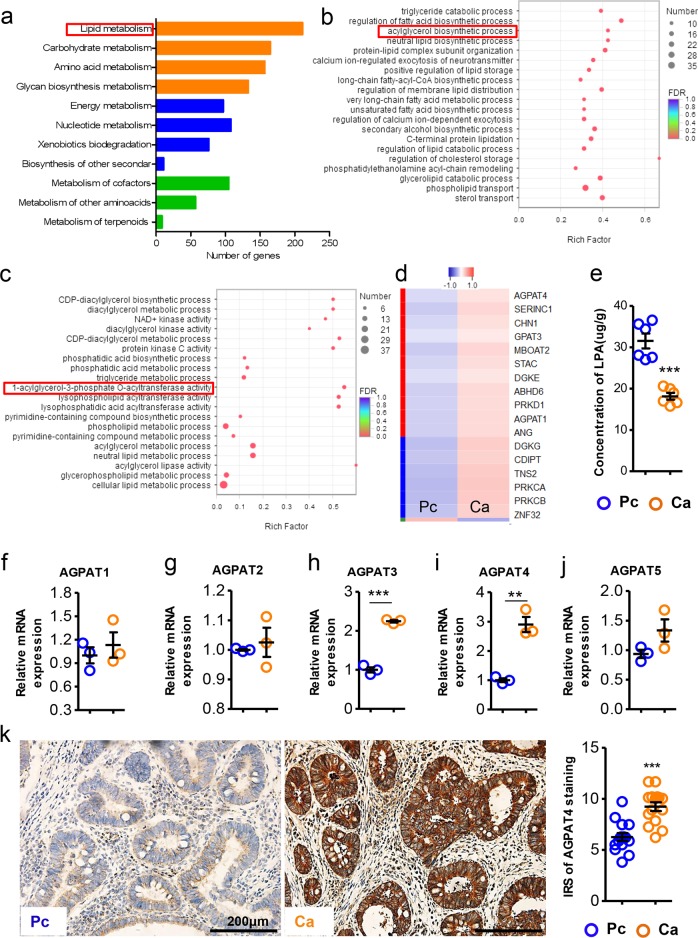
Aberrant upregulation of Agpat4 in colorectal cancer cells. **a**–**c** Paracarcinoma (Pc) and carcinoma (Ca) tissues were collected from patients with colorectal cancer (CRC) and subjected to RNA-sequencing analysis. Each tested sample was pooled from three individual samples. KEGG (**a**) or GO (**b, c**) pathway enrichment was performed as indicated. The pathways marked by red boxes were focused on in subsequent analyses. **b**, **c** The vertical axis indicates the GO term, and the horizontal axis indicates the Rich factor, which is defined as the ratio of enriched gene number to background gene number. The size of the circles indicates the gene number in the GO term, and the color of the circles indicates the false discovery rate (FDR)-corrected *P* value. **d** Heat map showing the relative expression of acylglycerol biosynthetic process-related genes in Pc and Ca tissues from CRC patients. **e** Concentrations of LPA in Pc and Ca tissues from CRC patients. (*n* = 6). **f**–**j** Relative mRNA levels of AGPAT1, AGPAT2, AGPAT3, AGPAT4, and AGPAT5 in the Pc and Ca tissues from CRC patients. **k** Representative images showing the immunostaining of AGPAT4 in Pa and Ca tissues from CRC patients. The statistical data were obtained by analyzing 15 pairs of samples from 15 patients. Scale bar represents 200 μm. (*n* = 15). The data in (**e**–**k**) represent the means ± s.e.ms. (**P < 0.01, ***P < 0.005; Student’s *t* test)

To investigate lipid metabolism in CRC, human CRC tissues and the corresponding paracarcinoma tissues were collected and subjected to RNA-sequencing analysis. The differentially expressed genes were enriched by KEGG or GO enrichment analyses. As shown in Fig. 1a, lipid metabolism was highly enriched according to KEGG pathway analysis. GO enrichment analysis showed that triglyceride catabolism and acylglycerol biosynthesis in CRC were obviously altered (Fig. 1b). We next focused on the acylglycerol biosynthetic process because triglyceride catabolism in CRC was intensively investigated in our previous studies.^14,18,19,24,25^ Further analysis of differentially expressed genes in the acylglycerol biosynthetic process indicated that phosphatidic acid metabolism-associated enzymes, especially AGPATs, were notably altered (Fig. 1c, d). AGPATs are responsible for converting lysophosphatidic acid (LPA) to phosphatidic acid.^26^ We found that LPA levels were decreased in human CRC tissues relative to those in paracarcinoma tissues (Fig. 1e), indicating an increase in AGPAT expression or activity. Using real-time PCR assays, we screened the expression of AGPAT family members and found that only AGPAT3 and AGPAT4 were upregulated in CRC tissues (Fig. 1f–j). For further confirmation, we obtained the expression data of AGPATs in primary CRC tissues and normal tissues from an online database (http://merav.wi.mit.edu/SearchByGenes.html). We also analyzed the relationship between AGPAT expression and survival rates of CRC patients in another online database (https://hgserver1.amc.nl/cgi-bin/r2/main.cgi). The upregulated expression of AGPAT4 in CRC tissues was confirmed in the online database, although the levels of AGPAT1, AGPAT2, AGPAT3, AGPAT4, and AGPAT5 in CRC tissues were differentially regulated (Fig. S1a–e). Notably, higher expression of AGPAT4 in the cancer tissues of CRC patients predicted worse survival (Fig. S1d). Given that LPA levels were reduced in CRC tissues, we concluded that AGPAT4, and not the other AGPATs, was the most important regulator of LPA production in CRC tissues. Indeed, immunohistochemical staining assays showed that AGPAT4 expression was higher in CRC tissues than in paracarcinoma tissues (Fig. 1k). In CRC patients, the expression of AGPAT4 in cancer tissues was positively correlated with the size and stages of primary tumors (Fig. S1f–j).

### Agpat4 silencing in CRC inhibits tumor progression

**Fig. 2:**
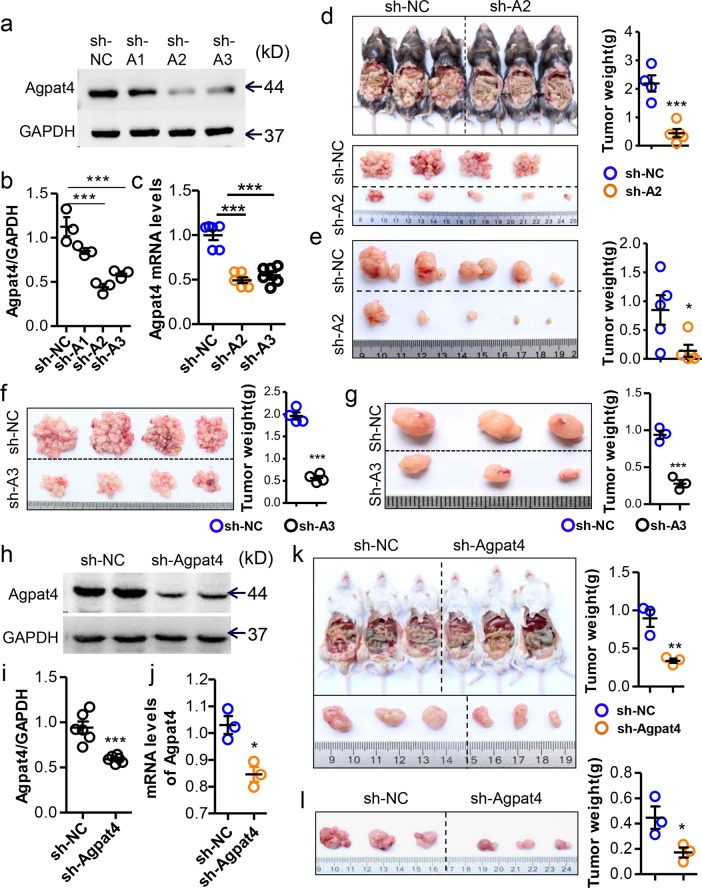
Agpat4 silencing in CRC inhibits tumor progression. **a** Immunoblotting assays of Agpat4 in MC-38 cells transfected with Agpat4 shRNAs (sh-A1, sh-A2, and sh-A3) or the PLKO vector as a control (sh-NC). **b** Statistical data showing the gray values in **a**. (*n* = 3). **c** Real-time PCR assays of Agpat4 in MC-38 cells transfected with sh-NC, sh-A2, or sh-A3. (*n* = 6). **d** Six-week-old male C57BL/6 mice were intraperitoneally inoculated with sh-A2- or sh-NC-transfected MC-38 cells (4 × 10^6^ cells in 100 μl of PBS for each mouse). Two weeks later, mice were sacrificed, and the tumor nodules were collected and displayed. (*n* = 4–5). **e** Six-week-old male C57BL/6 mice were subcutaneously injected with sh-A2- or sh-NC-transfected MC-38 cells (4 × 10^6^ cells in 100 μl of PBS for each mouse) for 15 days. Then, the mice were sacrificed, and the tumors were collected. (*n* = 5). **f** Six-week-old male C57BL/6 mice were intraperitoneally inoculated with sh-A3- or sh-NC-transfected MC-38 cells (4 × 10^6^ cells in 100 μl of PBS for each mouse). Two weeks later, the mice were sacrificed, and the tumor nodules were collected and displayed. (*n* = 4). **g** Six-week-old male C57BL/6 mice were subcutaneously engrafted with sh-A3- or sh-NC-transfected MC-38 cells (4 × 10^6^ cells in 100 μl of PBS for each mouse) for 15 days. Then, the mice were sacrificed, and the tumors were collected. (*n* = 3). **h** Immunoblotting assays of Agpat4 in CT-26 cells transfected with sh-A2 or sh-NC. sh-A2 was used for Agpat4 silencing (sh-Agpat4) in the subsequent experiments. **i** Statistical data showing the gray values in **f**. (*n* = 6). **j** mRNA levels of Agpat4 in CT-26 cells transfected with sh-Agpat4 or sh-NC. (*n* = 3). **k** Six-week-old male BALB/c mice were intraperitoneally injected with sh-Agpat4- or sh-NC-transfected CT-26 cells (4.0 × 10^6^ cells in 100 μl of PBS) for 2 weeks, and then tumor nodules were collected and weighed. (*n* = 3). **l** Six-week-old male BALB/c mice were subcutaneously inoculated with CT-26 cells (4.0 × 10^6^ cells per mouse) for 15 days. Tumor nodules were isolated and weighed. (*n* = 3). Data in **b**–**g** and **i**–**l** represent the means ± s.e.ms. (*P < 0.05, **P < 0.01, ***P < 0.005; Student’s *t* test)

To determine the role of Agpat4 in CRC progression, Agpat4 was silenced in the CRC cell line MC-38 by lentivirus-mediated shRNAs (Fig. 2a–c). Agpat4 silencing obviously inhibited the growth of MC-38 tumors in peritoneal and subcutaneous xenograft models (Fig. 2d–g). In addition, we knocked down Agpat4 expression in another CRC cell line, CT-26 (Fig. 2h–j). In line with the role of Agpat4 in MC-38 tumors, Agpat4 deficiency markedly suppressed CT-26 tumor progression in the peritoneal (Fig. 2k) and subcutaneous xenograft models (Fig. 2l). Unexpectedly, Agpat4 silencing did not affect the viability (Fig. S2a), apoptosis (Fig. S2b), cell cycle progression (Fig. S2c), or migration (Fig. S2d) of MC-38 cells in vitro. These results indicated that Agpat4 might affect CRC progression through TME modulation.

### Agpat4 silencing in CRC inhibits T cell-dependent tumor progression

**Fig. 3:**
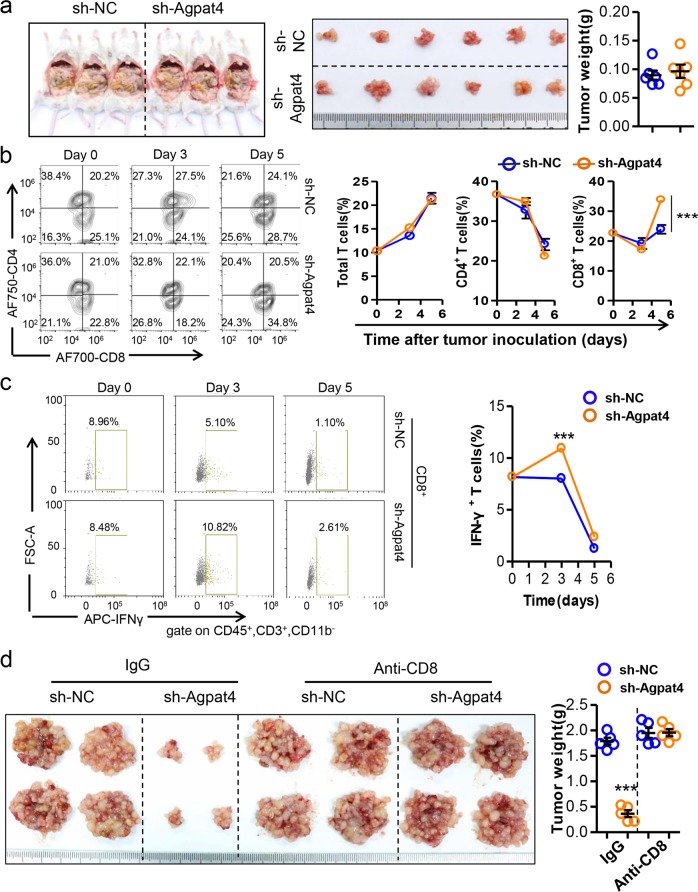
Agpat4 silencing in CRC suppresses T cell-dependent tumor progression. **a** Eight-week-old male NCG mice were intraperitoneally inoculated with sh-Agpat4- or sh-NC-transfected MC-38 cells (4 × 10^6^ cells in 100 μl of PBS per mouse). Twelve days later, the mice were sacrificed, and the tumor nodules were collected and weighed. (*n* = 6). **b** Agpat4-silenced MC-38 cells increased the percentage of CD8^+^ T cells in epididymal fats. Six-week-old mice were intraperitoneally engrafted with sh-Agpat4- or sh-NC-transfected MC-38 cells (4 × 10^6^ cells in 100 μl of PBS per mouse) on day 0 and were sacrificed for the analysis of total T cells, CD4^+^ T cells and CD8^+^ T cells in the stromal vascular fraction (SVF) of epididymal fats on days 0, 3, and 5. T cells were marked by the phenotype CD45^+^CD3^+^CD11b^−^. CD4^+^ T cells were defined by the phenotype CD45^+^CD3^+^CD11b^−^CD8^−^CD4^+^. CD8^+^ T cells were defined by the phenotype CD45^+^CD3^+^CD11b^−^CD4^−^CD8^+^. Representative results are shown. **c** Percentage of IFN-γ^+^ cells in the CD8^+^ T cells described above in **b**. (*n* = 5). **d** Six-week-old male C57BL/6 mice were intraperitoneally inoculated with MC-38 cells (4 × 10^6^ cells in 100 μl of PBS per mouse) on day 0 and then treated with anti-CD8 antibody or IgG as a control 3 times (days 2, 4, and 6) at a dose of 100 μg/day. Mice were sacrificed for tumor observation on day 14. (*n* = 5). The data in **a**–**d** represent the means ± s.e.ms. (***P < 0.005; Student’s *t* test)

The immune microenvironment is an essential factor affecting cancer progression.^6^ To observe whether changes in immunity were involved in Agpat4-regulated CRC progression, NCG mice with severe immune deficiency were used to establish xenograft models. Interestingly, we found that the tumor suppression induced by Agpat4 silencing disappeared in the context of immune deficiency (Fig. 3a). We next established peritoneal carcinomatosis models in immunocompetent mice and found that the number of CD8^+^ T cells was increased by Agpat4-deficient MC-38 cells (Figs. 3b and S3a). Moreover, Agpat4 deficiency in MC-38 cells increased the ratio of IFN-γ-producing CD8^+^ and CD4^+^ T cells (Figs. 3c and S3b, c). Similarly, the tumor suppression induced by Agpat4 silencing in immunocompetent mice was fully prevented by anti-CD8 or anti-CD4 neutralizing antibodies (Figs. 3d and S3d).

### Agpat4 silencing in CRC inhibits macrophage-dependent T-cell activation and tumor progression

**Fig. 4:**
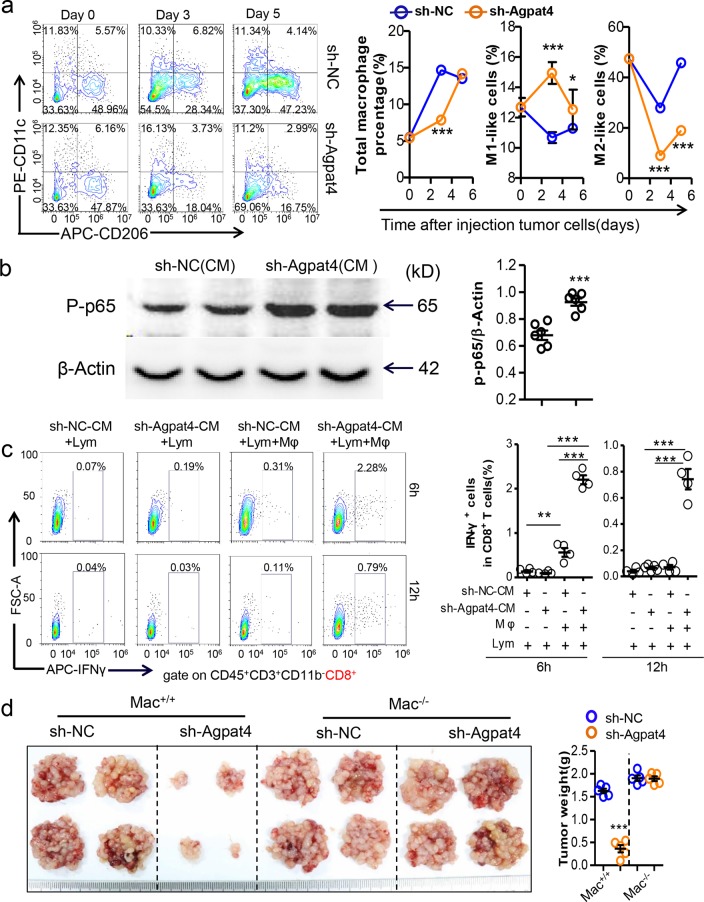
Agpat4 silencing in CRC suppresses macrophage-dependent T-cell activation and tumor progression. **a** Six-week-old mice were intraperitoneally inoculated with sh-Agpat4- or sh-NC-transfected MC-38 cells (4 × 10^6^ cells in 100 μl of PBS per mouse) on day 0 and were sacrificed for the analysis of total macrophages, M1 macrophages and M2 macrophages in the SVF of epididymal fats on days 0, 3, and 5. Macrophages were marked by the phenotype CD45R^−^CD90.2^−^Gr1^−^CD45^+^F4/80^+^. M1-like cells were defined by the phenotype CD45R^−^CD90.2^−^Gr1^−^CD45^+^F4/80^+^CD206^-^CD11c^+^. M2-like cells were marked by the phenotype CD45R^−^CD90.2^−^Gr1^−^CD45^+^F4/80^+^CD11c^−^CD206^+^. **b** Immunoblotting assays of phosphorylated p65 (P-p65) in PMs treated with conditioned medium (CM) from sh-NC- or sh-Agpat4-transfected MC-38 cells for 12 h. This experiment was repeated in triplicate. The gray values of the blotting were calculated. (*n* = 3). **c** Spleen lymphocytes cocultured with or without macrophages were treated with CM from sh-NC- or sh-Agpat4-transfected MC-38 cells for 6 or 12 h, and then the percentage of IFN-γ^+^ cells in CD8^+^ T cells was measured by flow cytometry. (*n* = 4). **d** Mac^+/+^ or Mac^−/−^ mice were intraperitoneally engrafted with sh-NC- or sh-Agpat4-transfected MC-38 cells (4 × 10^6^ cells in 100 μl of PBS per mouse). The tumor-bearing mice were sacrificed on day 15, and tumor nodules were collected. (*n* = 5). All data represent the means ± s.e.ms. (*P < 0.05, **P < 0.01, ***P < 0.005; Student’s *t* test)

Both innate and adaptive immunity are reeducated in the TME.^19,27,28^ Interactions between T cells and macrophages are associated with cancer progression. Therefore, we next observed whether the activity of macrophages in the TME was influenced by Agpat4 silencing in CRC cells. The results showed that Agpat4 knockdown in MC-38 cells mildly regulated macrophage infiltration at the early stage of peritoneal carcinomatosis. However, Agpat4 deficiency obviously increased the frequency of M1-like macrophages and decreased the frequency of the M2-like population (Figs. 4a and S4a). These results indicated that Agpat4 could regulate macrophage polarization in CRC. As expected, the conditioned medium (CM) from Agpat4-deficient MC-38 cells (sh-Agpat-CM) obviously stimulated the phosphorylation of p65 (Fig. 4b) and JNK (Fig. S4b), which are frequently activated in M1 macrophages. In addition, treatment with sh-Agpat-CM for 12 h (but not for 24 h) upregulated the IL-1β, IL-10, and Arg-1 mRNA levels (Fig. S4c). Furthermore, we found that the CM of MC-38 cells did not directly stimulate IFN-γ production in CD8^+^ T cells (Fig. 4c). In fact, the CM of MC-38 cells activated CD8^+^ T cells depending on the presence of macrophages, and sh-Agpat-CM had a more potent effect on stimulating IFN-γ production in CD8^+^ T cells than did the control CM (Fig. 4c). In line with the results in CD8^+^ T cells, similar conclusions were obtained in CD4^+^ T cells (Fig. S4d). To further validate the effect of macrophages on Agpat4 silencing-induced tumor suppression, macrophage-deficient (Mac^−/−^) mice were used to establish a colorectal peritoneal carcinomatosis model. The results showed that the Agpat4 silencing-mediated suppression of CRC growth was fully prevented by macrophage ablation (Fig. 4d).

### The Agpat4/LPA axis in CRC regulates macrophage activation and tumor progression

**Fig. 5:**
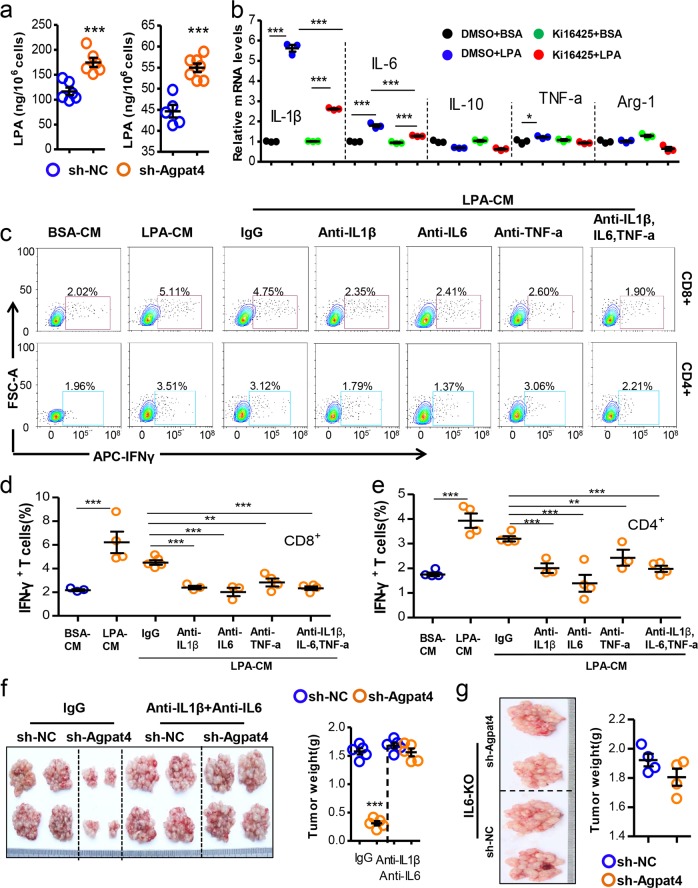
Agpat4/LPA axis in CRC regulates macrophage-dependent T-cell activation and cancer progression. **a** Intracellular (left) or supernatant (right) concentrations of LPA in sh-NC- or sh-Agpat4-transfected MC-38 cells. **b** mRNA levels of cytokines in peritoneal macrophages (PMs) treated with DMSO or Ki16425 (10 μM) plus BSA (1%) or LPA (200 μM) for 12 h. **c**–**e** CM of PMs treated with LPA (200 μM) or 1% BSA as a control for 12 h were collected and supplemented with anti-IL1β (10 μg/ml), anti-IL-6 (10 μg/ml), and/or anti-TNFα (10 μg/ml) antibodies for the treatment of spleen lymphocytes. The percentage of IFN-γ^+^ cells in CD4^+^ or CD8^+^ T cells was detected by flow cytometry analysis. **f** Six-week-old male mice were intraperitoneally injected with sh-Agpat4- or sh-NC-transfected MC-38 cells on day 0 and were treated with IL1β plus IL-6 antibodies or IgG as a control 3 times (days 2, 4, and 6) at a dose of 100 μg/day. Mice were sacrificed for tumor observation on day 15. (*n* = 5). **g** Six-week-old male IL-6-KO mice were intraperitoneally inoculated with sh-Agpat4- or sh-NC-transfected MC-38 cells on day 0. Tumor nodules were observed on day 15. All data represent the means ± s.e.ms. (*P < 0.05, **P < 0.01, ***P < 0.005; Student’s *t* test)

The aforementioned results indicated that sh-Agpat-CM contained some factors that might stimulate macrophage activation. We did not observe any obvious differences in the expression of inflammatory cytokines between the control and Agpat4-silenced MC-38 cells according to RNA-sequencing assays (data not shown). Therefore, we first considered whether Agpat4-regulated metabolites could affect macrophage activity. We found that Agpat4 silencing obviously increased the levels of LPA in cells and supernatants (Fig. 5a). LPA treatment for 12 or 24 h largely increased the mRNA levels of proinflammatory cytokines (IL-1β, IL-6, and TNFα) in peritoneal macrophages (PMs) (Fig. S5a, b), whereas this effect was largely prevented by inhibitors of LPA receptors 1 and 3 (Lpar1/3) (Fig. 5b). For confirmation, we silenced Lpar1/3 with siRNAs and found that LPA-stimulated IL-1β mRNA expression to a large extent through Lpar1/3 in PMs (Fig. S5c–e).

We next investigated whether LPA could affect T-cell activation. The results showed that LPA did not directly influence IFN-γ mRNA or protein levels in T cells (Fig. S5f, g). CM from LPA-primed macrophages obviously induced IFN-γ production in CD8^+^ and CD4^+^ T cells, whereas this effect was prevented by additional treatment with neutralizing antibodies anti-IL1β, anti-IL-6, and anti-TNFα or the three antibodies together (Fig. 5d, e). These results indicated that LPA-induced M1-like macrophage-dependent T-cell activation. Furthermore, we inactivated macrophages by deleting IL-6 expression (IL-6-KO) in macrophages. We found that CM from LPA-primed IL-6-knockout (IL-6-KO) macrophages could not stimulate IFN-γ production in CD8^+^ or CD4^+^ T cells (Fig. S5h). In the peritoneal carcinomatosis model, the Agpat4 silencing-induced suppression of CRC progression was fully prevented by the combined treatment with antibodies against IL1β and IL-6 (Fig. 5f). In addition, the Agpat4 silencing-induced suppression of CRC progression was largely abolished when a peritoneal xenograft model was established in IL-6-KO mice (Fig. 5g). These results indicated that M1-like activation marked by increased IL1β and IL-6 was required for LPA-induced T-cell activation and tumor suppression.

To correlate AGPAT4 expression with the infiltration and activation of macrophages and T cells in the TME of CRC patients, fresh CRC samples were collected and classified into AGPAT4^low^ and AGPAT4^high^ groups according to AGPAT4 expression in CRC tissues. The infiltration of macrophages, T cells, and their subpopulations was determined by flow cytometry analysis. The results showed that the AGPAT4^high^ group had less infiltration of total and M1-like macrophages than the AGPAT4^low^ group (Fig. S6a, b). Simultaneously, the ratios of CD8^+^, CD4^+^, and IFNγ^+^ T cells were decreased in the AGPAT4^high^ group relative to the respective values in AGPAT4^low^ tissues (Fig. S6c–e).

### The Agpat4/LPA axis in CRC regulates macrophage activation via the p38/p65 pathways

**Fig. 6:**
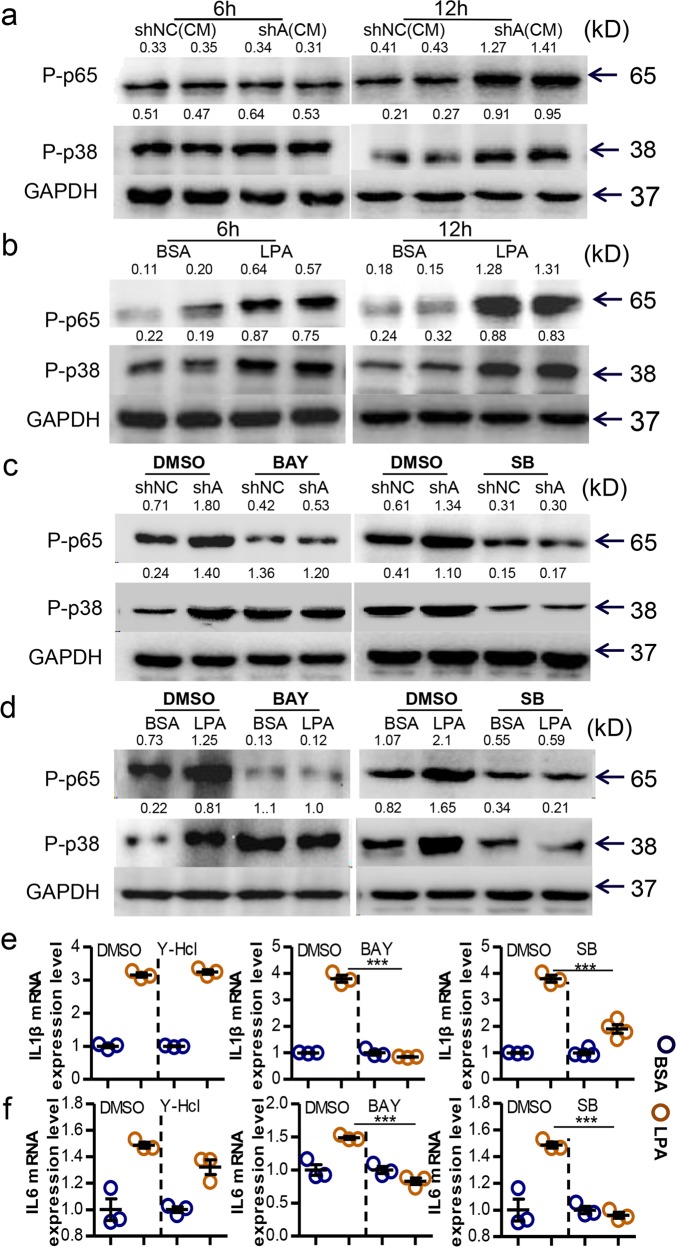
Agpat4/LPA axis in CRC regulates p38/p65 pathways and downstream cytokines in macrophages. **a** Immunoblotting assays of P-p65 and P-p38 in PMs treated with CM from sh-Agpat4- or sh-NC-transfected MC-38 cells for 6 h (left) or 12 h (right). **b** Immunoblotting assays of P-p65 and P-p38 in PMs treated with LPA (200 μM) or 1% BSA for 6 h (left) or 12 h (right). **c** PMs were pretreated with the NF-κB inhibitor BAY 11-7082 (10 μM) or the p38 inhibitor SB203580 (10 µM) for 4 h and then additionally stimulated with CM from sh-Agpat4- or sh-NC-transfected MC-38 cells for 12 h before being harvested for immunoblotting assays. **d** PMs were pretreated with the NF-κB inhibitor BAY 11-7082 (10 μM) or the p38 inhibitor SB203580 (10 μM) for 4 h and then additionally stimulated with LPA (200 μM) or 1% BSA for 12 h before being harvested for immunoblotting assays. **e**, **f** PMs were primed with BAY 11-7082 (10 μM), SB203580 (10 μM), or the ROCK inhibitor Y-HCL (10 µM) for 4 h, and then additionally treated with LPA (200 μM) or 1% BSA for 12 h before being harvested for real-time PCR assays of IL-1β (**e**) and IL-6 (**f**) levels. The data in **a**–**d** show the gray values for each band relative to that of GAPDH. The data in **e**, **f** show the means ± s.e.ms. (***P < 0.005; Student’s *t* test)

We next investigated how LPA stimulated macrophage activation. As previously reported, p65, p38, and ATF2 signaling are activated downstream of LPA receptors.^29–32^ As expected, we found that sh-Agpat-CM or LPA obviously stimulated the phosphorylation of p65 and p38 in PMs (Fig. 6a, b). However, sh-Agpat-CM or LPA did not affect the phosphorylation of ATF2 (Fig. S7a, b). Notably, sh-Agpat-CM- or LPA-induced p65 phosphorylation in macrophages was prevented by a p38 inhibitor, whereas sh-Agpat-CM- or LPA-stimulated p38 phosphorylation was not prevented by an NF-κB inhibitor (Fig. 6c, d), indicating that p65 was downstream of p38 signaling in LPA-treated macrophages. Similarly, the LPA-mediated increase in IL1β and IL-6 mRNA levels in macrophages was fully prevented by p38 or NF-κB inhibitors (Figs. 6e, f and S7c, d) but not by an inhibitor of Rock, which was previously reported to be activated by LPA.^33^

### The Agpat4/LPA/p38/p65 axis modulates CRC progression

**Fig. 7:**
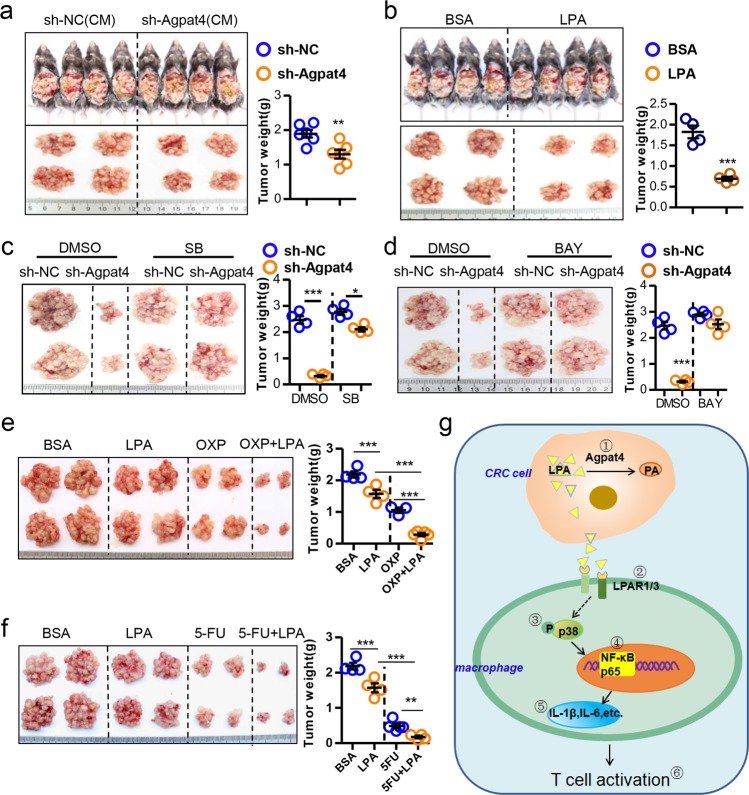
Agpat4/LPA/p38/p65 axis modulates CRC progression. **a** Six-week-old male C57BL/6 mice were intraperitoneally inoculated with MC-38 cells (4 × 10^6^ cells in 100 μl of PBS per mouse) on day 0 and then treated with CM from sh-Agpat4- or sh-NC-transfected MC-38 cells on days 1, 3, 5, 7, and 9. Mice were sacrificed on day 15, and the tumor nodules were weighed. (*n* = 6). **b** Six-week-old male C57BL/6 mice were intraperitoneally engrafted with MC-38 cells (4 × 10^6^ cells in 100 μl of PBS per mouse) on day 0 and then treated with LPA (50 mg/kg) or BSA as a control on days 1, 3, 5, 7, and 9. Mice were sacrificed on day 15, and the tumor nodules were observed. (*n* = 4). **c**, **d** Six-week-old male C57BL/6 mice were intraperitoneally injected with sh-Agpat4- or sh-NC-transfected MC-38 cells (4 × 10^6^ cells in 100 μl of PBS per mouse) on day 0 and then administered the inhibitors SB (25 mg/kg) (**c**) or BAY (20 mg/kg) (**d**) on days 1, 4, and 7. Fifteen days later, tumor nodules were displayed and weighed. (*n* = 4). **e** Six-week-old male C57BL/6 mice were intraperitoneally injected with MC-38 cells (4 × 10^6^ cells in 100 μl of PBS per mouse) on day 0 and then administered LPA (50 mg/kg) and/or oxaliplatin (OXP, 5 mg/kg) on days 1, 4, and 7. Fifteen days later, tumor nodules were observed. (*n* = 4). **f** Six-week-old male C57BL/6 mice were intraperitoneally inoculated with MC-38 cells (4 × 10^6^ cells in 100 μl PBS per mouse) on day 0 and then administered LPA (50 mg/kg) and/or 5-fluorouracil (5-FU, 50 mg/kg) on days 1, 4, and 7. Fifteen days later, tumor nodules were observed. (*n* = 4). **g** Proposed hypothesis for the mechanism by which the Agpat4/LPA axis regulating macrophage and T-cell activation in cancer. Agpat4-regulated production of LPA from CRC cells and signaling through the LPAR1/3 and p38/p65 pathways in macrophages stimulate M1 macrophage and T-cell activation. Aberrant upregulation of Agpat4 results in a reduction in LPA from CRC cells and suppression of antitumor immunity. The serial numbers ①–⑥ indicate the effective targets for CRC therapy in our study. The data in **a–f** show the means ± s.e.ms. (*P < 0.05, **P < 0.01, ***P < 0.005; Student’s *t* test)

We next validated the aforementioned mechanisms in a colorectal peritoneal carcinomatosis model. An MC-38 cell-based peritoneal carcinomatosis model was established in WT mice, and tumor growth was suppressed by treatment with sh-Agpat-CM (Fig. 7a) or LPA (Fig. 7b). The inhibitory effect of LPA on peritoneal carcinomatosis disappeared largely in the IL-6-KO mice (Fig. S8). We also demonstrated that the Agpat4 silencing-induced suppression of MC-38 tumor growth was fully prevented by treatment with p38 or NF-κB inhibitors (Fig. 7c, d). To further test the translational potential of LPA in CRC therapy, experiments with combined administration of LPA and regular chemotherapy drugs for CRC patients were carried out in the MC-38-based peritoneal carcinomatosis model. The results showed that LPA plus oxaliplatin or 5-fluorouracil synergistically inhibited CRC progression (Fig. 7e, f).

## Conclusions

Agpat4-regulated production of LPA from CRC cells signals through the LPAR1/3 and p38/p65 pathways in macrophages to stimulate M1-dependent T-cell activation and cancer suppression (Fig. 7g). Our findings might provide multiple targets for CRC therapy.

## Discussion

Lipid metabolic reprogramming is causally linked to the establishment of a protumor TME and the progression of CRC.^9,11^ However, how the LPA metabolic pathway is regulated in CRC cells and its role in the immune alteration of the TME have not been elucidated. Here, we found that AGPAT4 was aberrantly expressed, which resulted in LPA reduction in CRC cells. Agpat4-regulated LPA release from CRC cells activated T cells through p38/p65 signaling-mediated M1 polarization of tumor-associated macrophages and subsequently triggered an antitumor response.

Our findings indicate that AGPAT4 is a major regulator of LPA in CRC cells. There are five AGPAT members: AGPAT1, AGPAT2, AGPAT3, AGPAT4, and AGPAT5.^34^ The expression of these members was differentially regulated in CRC cells. Only Agpat4 was validated to regulate CRC progression in mouse models and was negatively correlated with survival rates in the present study. Agpat4 silencing represented an effective strategy for CRC therapy in our experimental models. Exploring Agpat4 inhibitors or therapeutic antibodies specific for AGPAT4 might have great potential in clinical translation.

LPA was previously reported to potentiate CRC progression, which seems contradictory to our present findings. It should be noted that we and others revealed completely different mechanisms for LPA-regulated CRC progression. Previous studies have mainly focused on the effects of LPA on the biological functions of CRC cells in vitro. They showed that LPA-induced cell proliferation in different cell lines through different receptors, suggesting that there is a cell line-specific difference in the relative importance of each LPA receptor in its ability to induce proliferation of CRC cells.^35,36^ LPA inhibited the motility of colon cancer cells through LPA receptors 4 and 6.^37^ Therefore, it is not surprising that Agpat4-regulated LPA did not obviously affect the proliferation, cell cycle progression, apoptosis, or migration of MC-38 cells in vitro in the present study. In contrast, we observed a robust suppression of CRC growth via LPA-modulated macrophage and T-cell activity in the TME.

Our results indicated that an effective antitumor response required not only CD8^+^ T cells but also CD4^+^ T cells because either CD4 or CD8 blockade could fully abolish the AGPAT4 deficiency-induced antitumor effect. We and others^38^ reported that the population of IFN-γ-producing CD4^+^ T cells was small in fat tissues but that its function should not be neglected. A small population of immune cells could exhibit a large effect in the early phase of cancer progression. Previous^39,40^ and recent^41^ studies have indicated that CD4^+^ T cells are required for antitumor responses. Our results indicated that the interaction between macrophages, T cells and their subpopulations was involved in the process of CRC development.

Interestingly, Agpat4 silencing-induced CRC suppression was stronger than that induced by LPA treatment in the present study. We think that Agpat4-deficient CRC cells could continuously release LPA and stimulate the antitumor response in the TME. However, a single treatment of LPA might have a short and limited effect on cancer suppression. To develop a more efficient therapy, multiple doses of LPA could be administered. It should be noted that combined therapy of nontoxic LPA and regular chemotherapy drugs could synergistically suppress CRC progression. These LPA-based therapies have great potential in clinical translation.

Overall, we demonstrated that the Agpat4/LPA axis in CRC cells regulated antitumor immunity via p38/p65 signaling in macrophages. Our findings might provide novel strategies for the management of CRC in the clinic.

## Materials and methods

### Cell culture

The mouse CRC cell lines MC-38 and CT-26 were maintained in our laboratory.^19,24^ All cells were authenticated and tested for mycoplasma. Primary macrophages and all the cell lines were cultured in Dulbecco’s Modified Eagle Medium (DMEM) supplemented with 10% fetal bovine serum (FBS) at 37 °C in a humidified 5% CO_2_ atmosphere.

### Mice

Mouse studies were approved by the Institutional Animal Care and Use Committee of Third Military Medical University (Army Medical University) and were carried out according to the “Guide for the Care and Use of Laboratory Animals” published by the US National Institutes of Health (publication no. 85–23, revised 1996). All the mice were housed in a pathogen-free facility with a 12-h light, 12-h dark cycle and were provided with food and purified water ad libitum. All wild-type (WT) mice on the C57BL/6 or BALB/c background were provided by the Animal Center of Third Military Medical University. NCG mice (NOD-Prkdc^em26Cd52^Il2rg^em26Cd22^/Nju) were purchased from the National Model Animal Resource Information Platform (#T001475, Nanjing University, China). IL-6-KO mice on the C57BL/6 background were obtained with the help of Cyagen Biosciences (Guangzhou, China). ROSA26-eGFP-DTA mice (#006331, Jackson Laboratory Stock) have a loxP-flanked STOP cassette preventing the expression of diphtheria toxin fragment A (DTA). Myeloid cell-specific deletion (Mac^−/−^) mice were generated by crossing ROSA26-eGFP-DTA mice with mice expressing lysozyme promoter-driven Cre recombinase (#004781, Jackson Laboratory Stock). The ROSA26-eGFP-DTA mice with lyz-Cre^−/−^ had normal myeloid cells marked as Mac^+/+^.

### Isolation of peritoneal macrophages (PMs)

Mice were intraperitoneally injected with 2 ml of 3% thioglycollate (#T9032, Sigma) on day 1 and euthanized with CO_2_ on day 3. Peritoneal cells were collected by washing with 5 ml of DMEM supplemented with 1% penicillin and streptomycin. After centrifugation, the peritoneal cells were resuspended and cultured in cell culture dishes. One hour later, all the floating cells were removed, and the attached cells were taken as PMs.

### Establishment of Agpat4-silenced MC-38 or CT-26 cell lines

The shRNAs targeting mouse Agpat4 were mediated by the PLKO.1 lentivirus packaging system provided by Hanbio Biotechnology (Shanghai, China). The shRNA sequences were as follows: sh-A1, 5′-CCGGGATGTGGTACTTCGTGGAAATCTCGAGATTTCCACGAAGTACCACATCTTTTTG-3′; sh-A2, 5′-CCGCACCAAAGGCTTTGCTATTACTTCAAGAGAGTAATAGCAAAGCCTTTGGTGTTTTTTG-3′; and sh-A3, 5′-CCGGCAACACTGCTGGGAGTCTTAACTCGAGTTAAGACTCCCAGCAGTGTTGTTTTTG-3′. The CRC cell line MC-38 was infected by each shRNA-containing lentivirus, and the most efficient shRNA, sh-A2 (hereafter referred to as sh-Agpat4), was selected for further experiments. Agpat4 expression in CT-26 cells was also silenced by the sh-A2 lentivirus. The cells infected with the empty lentivirus vector were used as a control (sh-NC). The establishment of stable cell lines was verified by immunoblotting and real-time PCR assays of the targets.

### Small interfering RNA (siRNA)-mediated gene silencing

To silence the expression of LPAR1 or LPAR3 in primary macrophages, siRNAs specific to those two genes were transfected into cells using LipoRNAiTM (#C0535, Beyotime, China) according to the manufacturer’s protocol. Briefly, macrophages were plated at a density of 2.5 × 10^5^ cells in a well of a six-well plate. At a confluence of 70%, transfection was carried out. A total of 125 μl of FBS-free DMEM supplemented with siRNA (100 pmol/ml) and LipoRNAi medium was maintained for 20 min at room temperature and then added to a six-well plate with 1.75 ml of normal DMEM. Twelve hours later, the medium was removed and replaced with complete growth medium for further culture. The sequences of the siRNAs targeting Lpar1 were as follows: lpar1-si1, 5′-GCUUCUACAAUGAGUCUAUTT-3′; lpar1-si2, 5′-CCAUUGUGAUGGGUGCUAUTT-3′; and lpar1-si3, 5′-CCGAGUUCAACUCUGCUAUTT-3′. The sequences of siRNAs targeting Lpar3 were as follows: lpar3-si1, 5′-GGAAGUUCCACUUUCCCUUTT-3′; lpar3-si2, 5′-GGAAUUGCCUCUGCAACAUTT-3′; and lpar3-si3, 5′-GCGGUAUACGUACGCAUCUTT-3′. The sequence of the scrambled control siRNA was as follows: 5′-UUCUCCGAACGUGUCACGUTT-3′.

### Enzyme-linked immunosorbent assay (ELISA)

The ELISA kit for LPA tests was purchased from R&D Systems. The detailed procedures were performed according to the manufacturer’s protocol (#CEK623Ge, USCN, China). Briefly, 50 μl of cell culture medium and 50 μl of detection reagent A were added into each well, incubated for 1 h at 37 °C and then washed with wash buffer three times. Then, 100 μl of detection reagent B was added to each well, followed by incubation at 37 °C for 30 min and washing with wash buffer three times. Finally, 90 μl of substrate solution was added to each well, and 50 μl of stop solution was supplemented 15 min later. The absorbance at 450 nm of each well was measured immediately.

### Western blotting assays

Cell proteins were extracted using RIPA lysis buffer (#P0013, Beyotime, China) supplemented with 1% protease inhibitor cocktail (#04693116001, Roche, USA) and 1 mM phosphatase inhibitors (#2850, Sigma). Protein concentration was quantified using a BCA kit (#P0010, Beyotime, China). Samples (15 μl per well) were separated by SDS-PAGE on a 10% gel and transferred to a polyvinylidene difluoride membrane. The membrane was blocked with 5% BSA at room temperature for 1 h and incubated with primary antibodies overnight at 4 °C. The membranes were rinsed five times with PBS containing 0.1% Tween 20 (PBST) and incubated with the appropriate horseradish peroxidase-conjugated secondary antibody at room temperature for 1 h. Membranes were then extensively washed three times with PBST. The signals were stimulated with Enhanced Chemiluminescence Substrate (#BG0001, Bioground, China) for 1 min and detected with a Bio-Rad ChemiDoc MP System (170-8280). The primary antibodies included anti-Agpat4 (#PA5-62768, Invitrogen, the dilution ratio was 1:1,000), anti-phospho-p65 (Ser536) (#3033, Cell Signaling, the dilution ratio was 1:1,000), anti-phospho-p38 MAPK (Thr180/Tyr182) (#4511, Cell Signaling, the dilution ratio was 1:1,000), anti-phospho-ATF2 (Thr71)/ATF-7 (Thr53) (#24329, Cell Signaling, the dilution ratio was 1:1,000), anti-phospho-SAPK/JNK (Thr183/Tyr185) (P-JNK) (#4668 S, Cell Signaling, the dilution ratio was 1:1,000), anti-GAPDH (#60004-1-lg, Proteintech, the dilution ratio was 1:1,000), and anti-β-actin (#66009-1-lg, Proteintech, the dilution ratio was 1:1,000). Images were cropped for presentation. Full-size images can be provided upon request.

### Real-time PCR

Total RNA was isolated using TRIzol reagent (#30237, CWBIO) according to the manufacturer’s protocol. Briefly, 1 μg of RNA was reverse transcribed into cDNA using the PrimerScript RT reagent kit (#RR037A, TaKaRa, Shiga, Japan). Quantitative PCR was carried out using an ABI 7500 Real-Time PCR system (Applied Biosystems, Darmstadt, Germany). Reactions were performed in duplicate using a TB Green premix kit (#RR820a, TaKaRa, Shiga, Japan). The mRNA levels were normalized to Gapdh or GAPDH. The relative expression was calculated by the 2^(−DDCt)^ method. The primers are displayed in Supplementary Table 1.

### Immunohistochemistry of patient samples

Tumor tissues were collected in compliance with the regulations approved by the Scientific Investigation Board of Southwest Hospital in Third Military Medical University. All CRC samples in this study were primary samples from patients who were untreated before surgery, and the specimens were anonymized. Samples were formalin-fixed and paraffin-embedded. The tissue slides were dewaxed and rehydrated, and antigens were retrieved with 1 × EDTA antigen retrieval solution (#C1034, Solarbio, China) for 5 min in a pressure cooker. H_2_O_2_ (3%) was added to inactivate endogenous enzyme activity for 10 min at room temperature. After specific blocking, the slides were incubated with anti-Agpat4 antibody (#PA5-62768, Invitrogen, the dilution ratio was 1:500) at 4 °C overnight. Then, the relevant secondary antibody was supplemented for incubation at room temperature for 10 min, and visualization with streptomyces antibiotin peroxidase solution was performed. Slides without treatment with the primary antibody served as negative controls. All slides were developed with the EnVisionTM method (DAKO, Capinteria, CA), visualized using diaminobenzidine solution, and then lightly counterstained with hematoxylin. Evaluation of the immunohistochemical staining reaction was performed in accordance with the immunoreactive score (IRS) proposed by Remmele and Stegner. IRS = SI (staining intensity) × PP (percentage of positive cells): Negative SI = 0; weak SI = 1; moderate SI = 2; and strong SI = 3; negative PP = 0; 10% PP = 1; 11–50% PP = 2; 51–80% PP = 3; and >80% PP = 4. Ten microscopic fields (×100) from different areas of each tissue section were used for the IRS evaluation.

### Mouse models of peritoneal carcinomatosis

A mouse model was used to investigate the peritoneal metastasis of cancer cells and was introduced in detail in our previous studies.^42^ Briefly, 6-week-old male C57BL/6 or BALB/c mice were intraperitoneally injected with MC-38 or CT-26 cells (4 × 10^6^ cells in 100 μl of PBS per mouse), respectively. The tumor-inoculated mice were sacrificed ~2 weeks later. The peritoneal tumor nodes were collected, and their weight was calculated. This study was approved by the Institutional Animal Care and Use Committee of Third Military Medical University and was performed in accordance with the relevant guidelines.

### Mouse models of subcutaneous tumor

A mouse model was established as described in our previous work.^19^ Briefly, 6-week-old male C57BL/6 or BALB/c mice were subcutaneously injected with MC-38 or CT-26 cells (4 × 10^6^ cells in 100 μl of PBS for each mouse) in the thigh, respectively. The tumor-bearing mice were sacrificed on days 14 or 18, and the tumor nodes were collected and weighed. This study was approved by the Institutional Animal Care and Use Committee of Third Military Medical University and was carried out in accordance with the relevant guidelines.

### Fluorescence-activated cell sorting (FACS) of mouse fat tissues

Mice were sacrificed under CO_2_. The epididymal fats from normal and tumor-bearing mice were resected into ~1 mm^3^ with ophthalmic forceps and then digested with 25 ml of DMEM supplemented with 1 mg/ml collagenase IV (A004186, Sangon Biotech, China) and 3% FBS at 37 °C for 45 min. During the incubation, the solution was vibrated by a magnetic stirrer. The dissociated cells were filtered through a 75-μm filter (F513442, Sangon Biotech, China) and collected into a 50-ml tube and centrifuged for 10 min at 500 g. Then, the cells were resuspended and lysed with ACK buffer (R1010, Solarbio, China) to remove erythrocytes. Finally, the cells were resuspended in FACS buffer (PBS with 0.5% endotoxin-free FBS, 2 mM EDTA, and 25 mM HEPES) and incubated with conjugated antibodies for 45 min at 4 °C, followed by two washes in FACS buffer. Cells were resuspended in FACS buffer for FACS analysis (FACSVerse C6, BD Biosciences). The antibodies included Alexa Fluor 700 anti-mouse CD45 (#103128, BioLegend) and anti-mouse CD8a (#100730, BioLegend); APC/Cy7 anti-mouse F4/80 (#123118, BioLegend) and anti-mouse CD4 (#1100414, BioLegend); PE anti-mouse CD11c (#117308, BioLegend) and anti-mouse CD3 (#100206, BioLegend); FITC anti-mouse CD11b (#101206, BioLegend); APC anti-mouse CD206 (#141708, BioLegend) and anti-mouse IFN-γ (#505810, BioLegend); PerCP/Cy5.5 anti-mouse CD45 (#103132, BioLegend), anti-mouse Ly-6G/Ly-6C (Gr-1) (#108428, BioLegend), anti-mouse CD45R (#103236, BioLegend), and anti-mouse CD90.2 (#105338, BioLegend); PE anti-mouse CD11c isotype control (#1400907, BioLegend); and APC anti-mouse CD206 isotype control (#400511, BioLegend) and anti-mouse IFN-γ isotype control (#400411, BioLegend). T cells were marked by CD45^+^CD3^+^CD11b-. CD4^+^ T cells were defined by the phenotype CD45^+^CD3^+^CD11b^−^CD8^−^CD4^+^. CD8^+^ T cells were defined by the phenotype CD45^+^CD3^+^CD11b^−^CD4^−^CD8^+^. Macrophages were marked by the phenotype CD45R^−^CD90.2^−^Gr1^−^CD45^+^F4/80^+^. M1-like cells were defined by the phenotype CD45R^−^CD90.2^−^Gr1^−^CD45^+^F4/80^+^CD206^−^CD11c^+^. M2-like cells were marked by the phenotype CD45R^−^CD90.2^−^Gr1^−^CD45^+^F4/80^+^CD11c^−^CD206^+^.

### Preparation of conditioned medium (CM) from MC-38 cells

sh-Agpat4- or sh-NC-transfected MC-38 cells were cultured in 250-ml flasks in regular medium. At 80% confluence, 10 ml of DMEM supplemented with 1% FBS was added to each flask and recollected 48 h later to obtain tumor-primed medium. The CM was obtained by mixing the tumor-primed medium with regular medium (v/v = 1:1). These CM were used to treat cultured macrophages in vitro or colorectal peritoneal carcinomatosis model mice.

### Preparation of CM from PMs

PMs were isolated and plated at 80% confluence in 250-ml flasks in regular medium. Ten milliliters of DMEM supplemented with 1% FBS or LPA (200 μM) was added and recollected 12 h later to obtain the LPA-primed CM. The BSA- or LPA-primed CM were supplemented with anti-IL1β (10 μg/ml), anti-IL-6 (10 μg/ml), and/or anti-TNFα (10 μg/ml) antibodies for the treatment of spleen lymphocytes.

### Treatment of antibodies in vivo

Six-week-old male C57BL/6 mice were intraperitoneally injected with sh-Agpat4- or sh-NC-transfected MC-38 cells (4.0 × 10^6^ cells in 0.1 ml of PBS per mouse). The tumor-bearing mice were treated with neutralizing antibodies, including anti-CD4 (#100401, BioLegend, China), anti-CD8a (#100775, BioLegend, China), anti-IL1-β (#503502, BioLegend, China), anti-IL-6 (#504506, BioLegend, China), and anti-TNFα (#506325, BioLegend, China) or IgG (#400413, BioLegend) as a control.

### Statistical analysis

All analyses were performed using GraphPad Prism 5 (GraphPad Software, Inc.). All data are expressed as the means ± s.e.ms. and were analyzed using a two-tailed unpaired Student’s *t* test. For each parameter of all data presented, *P < 0.05, **P < 0.01, and ***P < 0.005.

## Supplementary information

Supplementary information

## Data Availability

All the data that support the findings of this study are available within the article and its supplementary information files or from the corresponding author upon reasonable request. The RNA-sequencing data will be deposited in the Gene Expression Omnibus.
