# Supplementary material for: The Agpat4/LPA axis in colorectal cancer cells regulates antitumor responses via p38/p65 signaling in macrophages
Source: Signal Transduct Target Ther. 2020 Mar 27;5:24. doi: 10.1038/s41392-020-0117-y (PMC7099097; doi:10.1038/s41392-020-0117-y)
Supplement: Supplementary file 1 — Supplementary information [file 41392_2020_117_MOESM1_ESM.docx]

Supplementary Materials for

The Agpat4/LPA axis in colorectal cancer cells regulates antitumor responses via p38/p65 signaling in macrophages

Dapeng Zhang^1,2^, Rongchen Shi^2^, Wei Xiang^2^, Xia Kang^2^, Bo Tang^3^, Chuan Li^3^, Linfeng Gao^3^, Xuan Zhang^4^, Lili Zhang^5^, Rongyang Dai^1*^, Hongming Miao^1,2*^

Correspondence to: hongmingmiao@sina.com; dryrun2502@163.com

**This PDF file includes:**

Materials and Methods

Figures. S1 to S8

Table S1

Materials and Methods

Cell viability assays

The CCK8 assays were performed according to the manufacturer’s protocol (#424501, BioLegend). The sh-NC- or sh-Agpat4-transfected MC-38 cells (2000 cells per 100 μl of medium) were plated into 96-well plates. At different time points, 10 μl of CCK8 solution was added to each well, and the cells were cultured at 37 °C in a humidified 5% CO_2_ atmosphere for 1 h. Then, the absorbance at 450 nm of each well was measured.

Wound healing tests

The sh-NC- or sh-Agpat4-transfected MC-38 cells were cultured in 12-well plates. At 80% confluence, uniformed wounds were created using a pipette to make a wound 500 μm wide. Being washed with PBS three times, the cells were cultured with FBS free DMEM media. Images of the scratches were captured at different time points using a digital camera (C5060, Olympus, Tokyo, Japan) mounted on an inverted microscope (CKX41, Olympus). Five different fields from each sample were considered for quantitative estimation of the distance between the borderlines, and in each image, three different equidistant points were measured to better estimate the true width of the wounded area. The migration rate was expressed as reduced percentage of the wound area calculated by the software Image J.

Cell cycle analysis

MC-38 cells were digested to single cells and washed twice with cold PBS before fixed with cold ethanol (75%) overnight. After washed with cold PBS, all cells were stained with propidium iodide (#1246MG010, BioFroxx, China) for 15 min before Flow cytometry analysis (FACSAria, BD Bioscience).

Flow cytometry assays of apoptosis

Cell apoptosis assays were performed by using the APC Annexin V Apoptosis Detection Kit with PI (#64093, BioLegend). The cells were trypsinized and washed with serum-containing medium. Cells were then centrifuged for 5 min at 1500 rpm and the supernatant was discarded. Then the cells were re-suspended with 100 μl Annexin binding buffer. 5 μl of Annexin V-APC and 5 μl of PI solutions were added to the cell suspension and incubated at 37 °C for 15 min. The stained cells were analyzed by Flow cytometry analysis (FACSAria, BD Bioscience).

Fluorescence-activated cell sorting (FACS) of human CRC tissues

The specimens of patients with CRC were conducted with permission from the Institutional Research Ethics Committee of Southwest Hospital in Third Military Medical University. The fresh CRC tumor tissues were cut into pieces and digested in FACS buffer supplemented with 1 g L^-1^ type 4 collagenase (#LS004188, Worthington), 0.1 g L^-1^ hyaluronidase (#H1115000, Sigma) and 0.01 g L^-1^ DNase Ⅰ(#D8071, Solarbio, China). The dissociated cells were collected into a 15-ml tube and centrifuged at 400×g for 5 min. The pellets were resuspended with ACK Lysing Buffer and washed with FACS buffer before filtration with a 75-μm filter. These cells were collected for further isolation of macrophages and T cells. The antibodies included PerCP/Cy5.5 anti-human CD45 antibody (#368504, BioLegend), APC anti-human CD68 antibody (#333810, BioLegend), PE anti-human CD11c antibody (#301605, BioLegend), PE anti-mouse CD11c isotype control antibody (#400111, BioLegend), Alesa Fluor 700 anti-human CD206 antibody (#321131, BioLegend), Alesa Fluor 700 anti-mouse CD206 isotype control antibody (#400143, BioLegend), PE anti-human CD3 antibody (#317308, BioLegend), APC/Cy7 anti-human CD4 antibody (#317418, BioLegend), FITC anti-human CD8 antibody (#344704, BioLegend), APC anti-human IFN-γ antibody (#502512, BioLegend). Macrophages were marked by CD45^+^CD68^+^. M1-like cells were assessed as CD45^+^CD68^+^CD206^-^CD11c^+^. M2-like cells were marked by CD45^+^CD68^+^CD11c^-^CD206^+^. T cells were marked by CD45^+^CD3^+^. CD4^+^ T cells were assessed as CD45^+^CD3^+^CD8^-^CD4^+^. CD8^+^ T cells were assessed as CD45^+^CD3^+^CD4^-^CD8^+^.

**
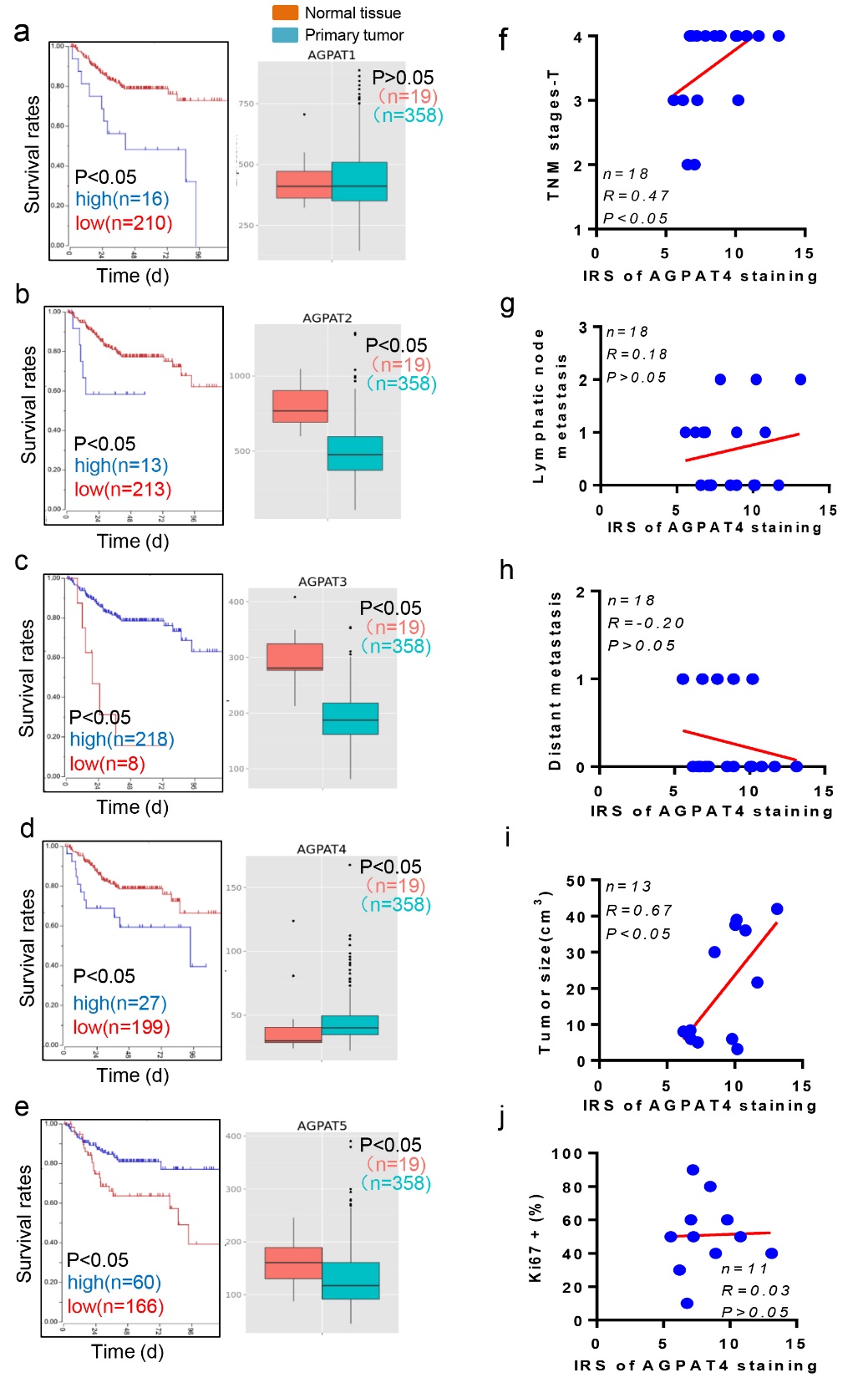
**

Figure. S1.

Correlation between AGPAT expression and survival rates of CRC patients in clinical data base

(**a-e**) The expression levels of AGPAT1, 2, 3, 4 and 5 in cancer tissues and their relationships with the survival rates in CRC patients. The sample sizes were indicated above. The expression data were collected from the data base: http://merav.wi.mit.edu/SearchByGenes.html. The survival rate data were from another data base: https://hgserver1.amc.nl/cgi-bin/r2/main.cgi. (Dataset: Tumor Colon-Sieber-290-MAS5.0-u133p2.) The survival rates were analyzed by Kaplan Meier survival analysis.

(**f-j**) The correlation between AGPAT4 expression and clinical pathological features in CRC patients. Expression of AGPAT4 in CRC tissues from 18 patients undergoing surgery was determined by immunohistochemical staining and IRS evaluation. The clinical pathological features including T stages (T2, T3 and T4), lymphatic node metastasis (N0, N1 and N2), distant metastasis (M0 and M1), tumor size and positive ratio of Ki67 were recorded. Linear regression analysis was performed to investigate the correlation. R value indicates the correlation coefficient. P<0.05 indicates the significance of difference.


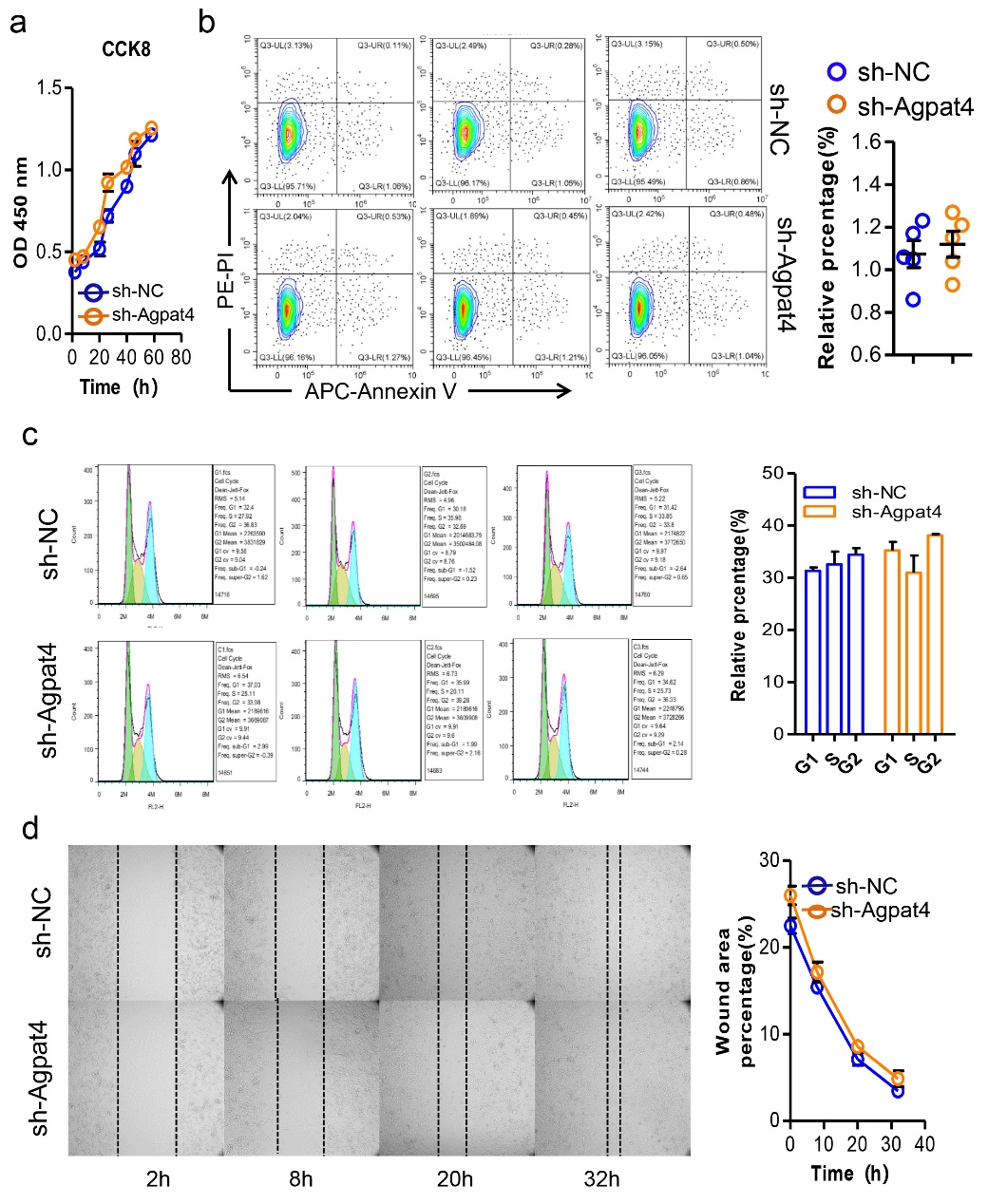


Figure. S2.

Agpat4 silencing does not affect the growth, apoptosis, cycle or migration of MC-38 cells in vitro

(**a**) CCK8 assays of the sh-NC- or sh-Agpat4-transfected MC-38 cells. (n=5)

(**b**) Apoptosis of sh-NC- or sh-Agpat4-transfected MC-38 cells was analyzed by flow cytometry. (n=5)

(**c**) Cell cycle of sh-NC- or sh-Agpat4-transfected MC-38 cells was analyzed by flow cytometry. (n=3)

(**d**) Wound healing assays of the sh-NC- or sh-Agpat4-transfected MC-38 cells. (n=3)

All data represent the means±s.e.ms, (P>0.05; Student’s *t*-test).


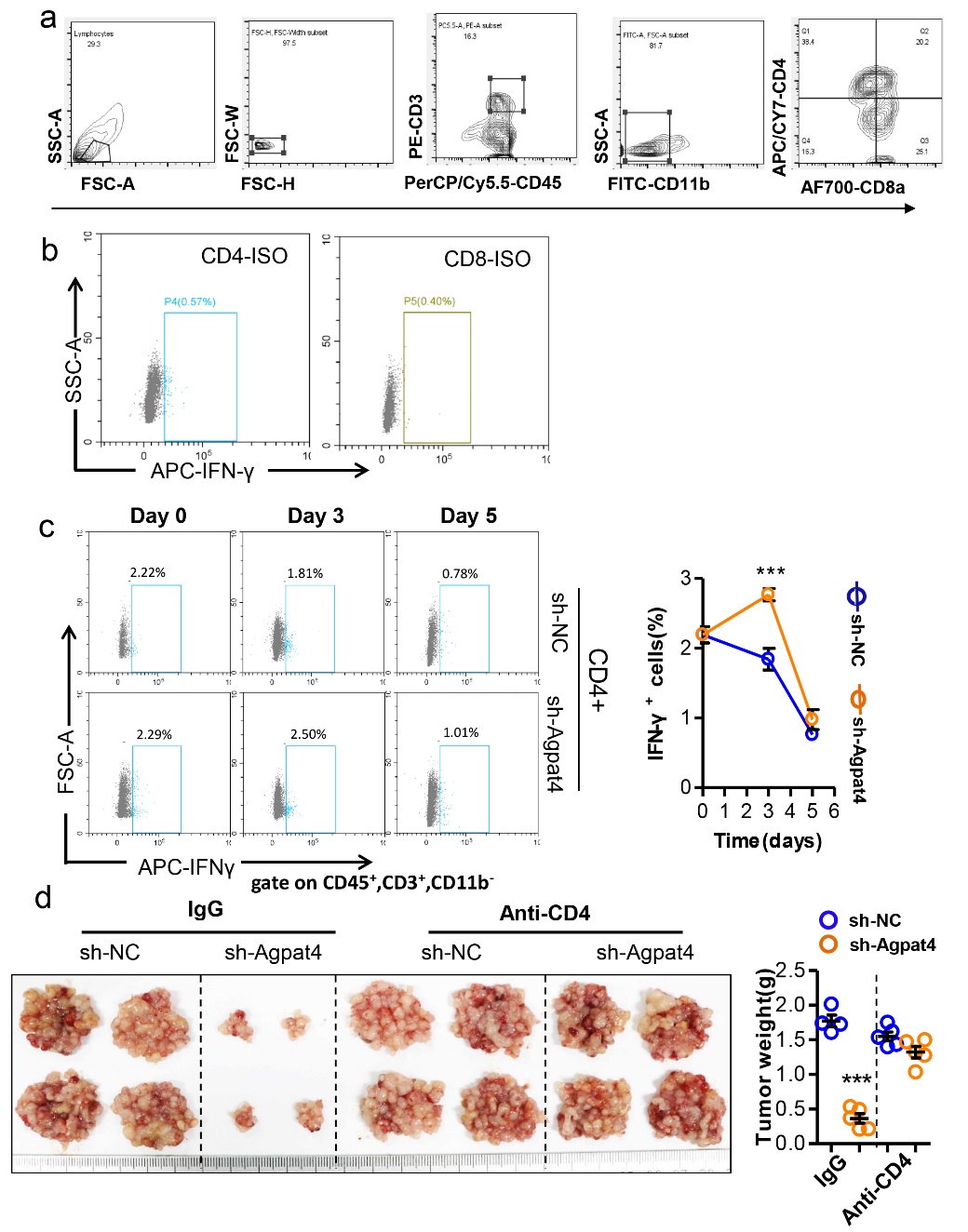


Figure. S3.

Agpat4 silencing inhibits CD4^+^ T cell-dependent CRC progression

(**a**) FACS gating strategy for T cells in epididymal fats. Debris and doublets were removed, and then the epididymal fat CD4^+^ T cells were assessed as CD45^+^CD3^+^CD11b^-^CD8^-^CD4^+^ and CD8^+^ T cells were marked by CD45^+^CD3^+^CD11b^-^CD4^-^CD8^+^.

(**b**) FACS gating strategy for measuring IFN-γ^+^ T cells in epididymal fats. IFN-γ^+^ T cells were gated on CD4^+^ or CD8^+^ T cells as described in (**a**).

(**c**) Percentage of IFN-γ^+^ cells in CD4^+^ T cells from epididymal fats. 6-week-old mice were intraperitoneally injected with sh-Agpat4- or sh-NC-transfected MC-38 cells (4×10^6^ cells in 100 μl PBS per mouse) on day 0 and were sacrificed for the analysis of IFN-γ^+^ T cells in the stromal vascular fraction (SVF) of epididymal fats on days 0, 3 and 5.

(**d**) Six-week-old male C57BL/6 mice were intraperitoneally inoculated with MC-38 cells (4×10^6^ cells in 100 μl PBS per mouse) on day 0, and then treated with anti-CD4 antibody or IgG as a control for 3 times (days 2, 4, and 6) at a dose of 100 μg/day. Mice were sacrificed for tumor observation on day 14. (n=5)

Data in (**c,d**) represent the means±s.e.ms. (***P<0.005; Student’s *t*-test).


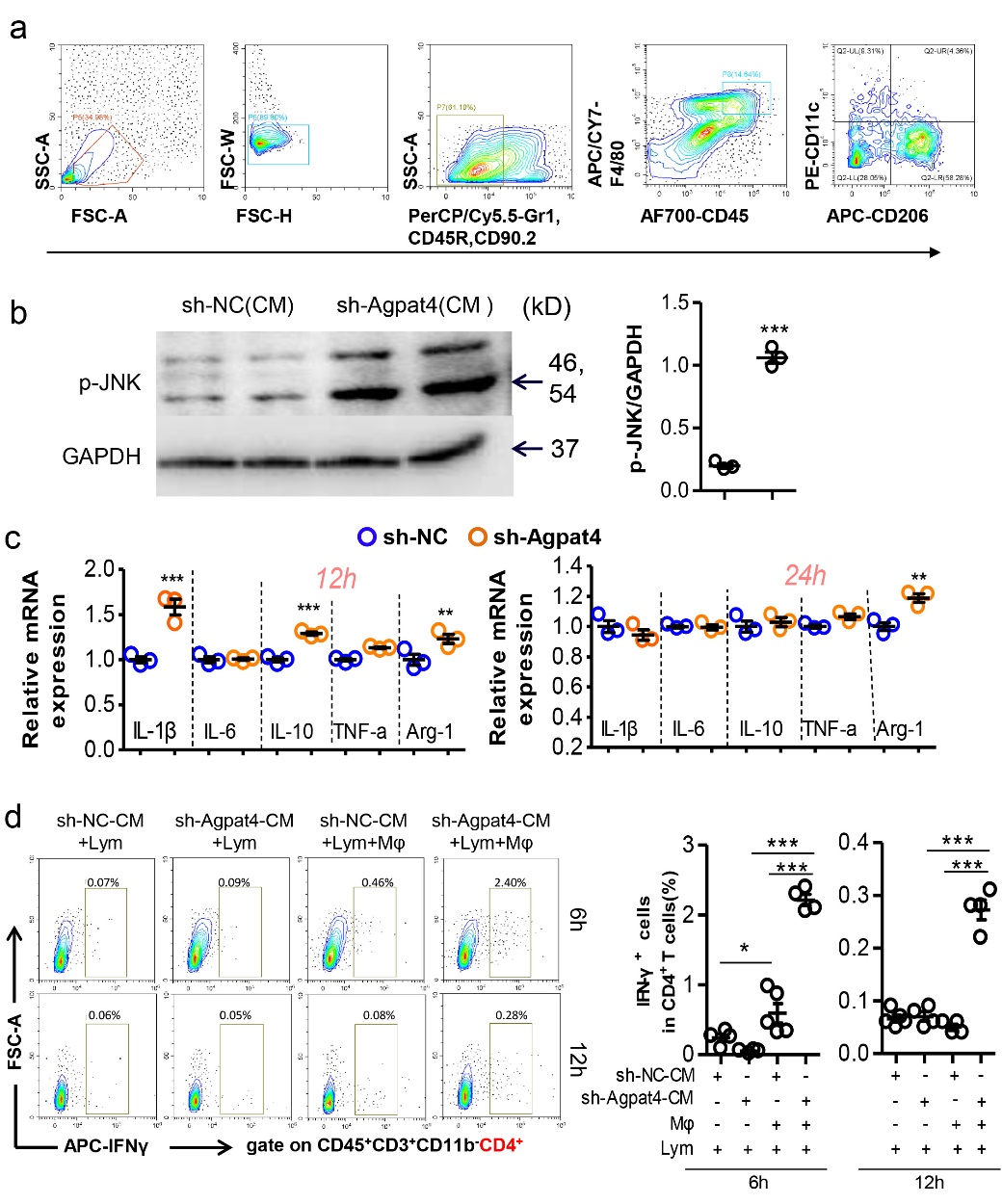


Figure. S4.

Agpat4 silencing inhibits CD4^+^ T cell-dependent CRC progression

(**a**) FACS gating strategy for macrophages in epididymal fats. Debris and doublets were removed, and then the SVFs were measured by flow cytometry. Macrophages were marked by CD45R^-^CD90.2^-^Gr1^-^CD45^+^F4/80^+^. M1-like cells were assessed as CD45R^-^CD90.2^-^Gr1^-^CD45^+^F4/80^+^CD206^-^CD11c^+^. M2-like cells were marked by CD45R^-^CD90.2^-^Gr1^-^CD45^+^F4/80^+^CD11c^-^CD206^+^.

(**b**) Immunoblotting assays of phosphorylated JNK (P-JNK) in peritoneal macrophages (PMs) treated with the CMs from sh-NC- or sh-Agpat4-transfected MC-38 cells for 12 h. This experiment was repeated in triplicate. Gray values of the blotting were calculated. (n=3)

(**c**) mRNA levels of cytokines in the PMs treated with CMs from sh-NC- or sh-Agpat4-transfected MC-38 cells for 12 h or 24 h.

(**d**) Spleen lymphocytes cocultured with or without macrophages were treated with CMs from the sh-NC- or sh-Agpat4-transfected MC-38 cells for 6 h or 12 h, and then the percentage of IFN-γ^+^ cells in CD4^+^ T cells was measured by flow cytometry. (n=4)

Data in (**b-d**) represent the means±s.e.ms. (*P<0.05, **P<0.01, ***P<0.005; Student’s *t*-test).


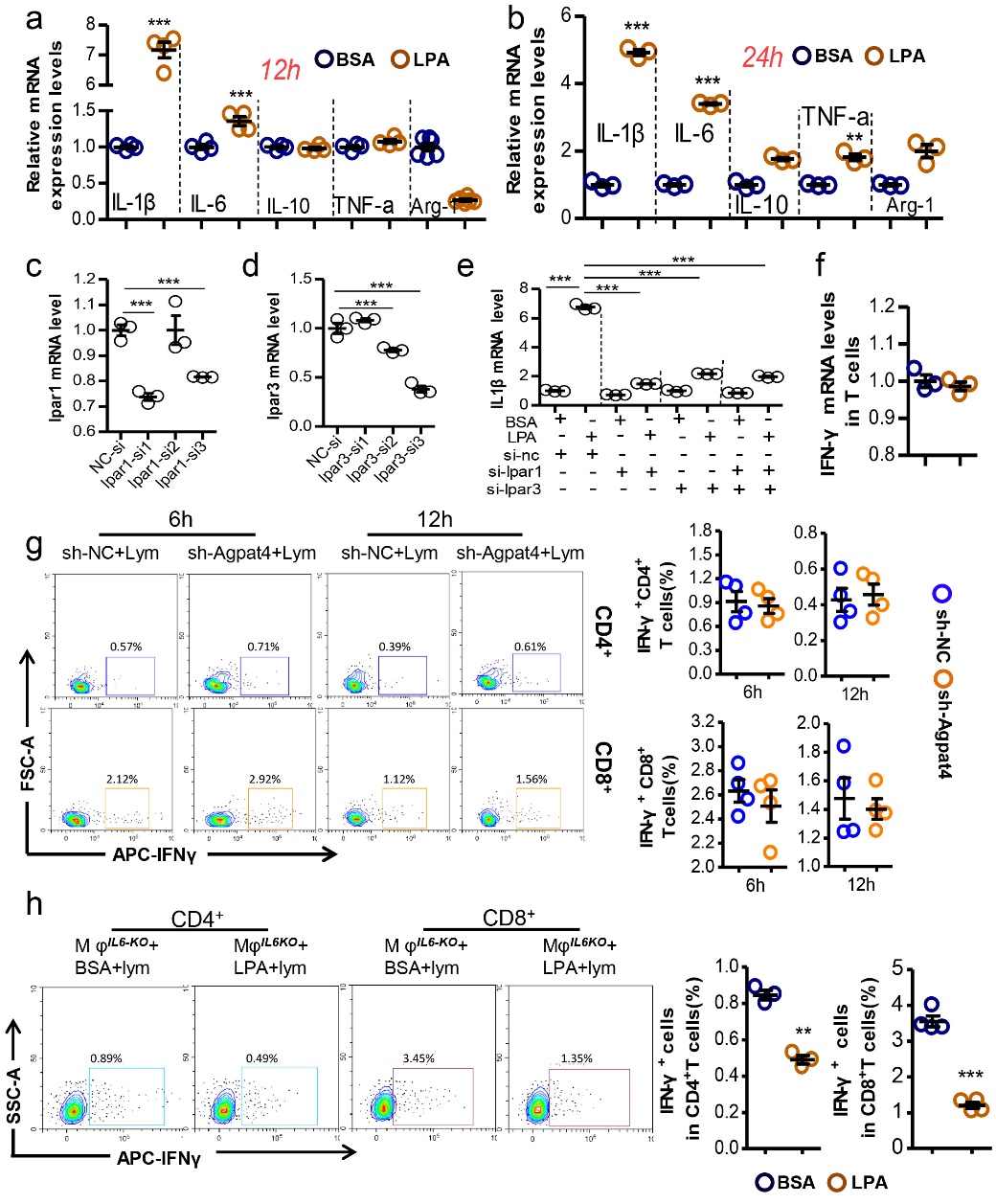


Figure. S5.

LPA stimulates T cell activity by activating macrophages through LPA1/3

(**a,b**) mRNA levels of cytokines in PMs treated with LPA (200 μM) or 1% BSA as control for 12 h (**a**) or 24h (**b**).

(**c**) mRNA levels of *lpar1* in PMs transfected with lpar1-si1, lpar1-si2, lpar1-si3 or NC-si as control for 24 h.

(**d**) mRNA levels of *lpar3* in PMs transfected with lpar3-si1, lpar3-si2, lpar3-si3 or NC-si as control for 24 h.

(**e**) PMs transfected with lpar1-si1 and/or lpar3-si3 were treated with LPA (200 μM) or 1% BSA as control for 24 h.

(**f**) IFN-γ mRNA levels in T cells treated with LPA (200 μM) or 1% BSA for 12 h.

(**g**) The sh-NC- or sh-Agpat4-transfected MC-38 cells were cocultured with lymphocytes from spleens for 6 h or 12 h. Then, the percentage of IFN-γ^+^ cells in CD4^+^ or CD8^+^ T cells was measured by flow cytometry analysis.

(**h**) The PMs from IL6-KO mice were treated with LPA (200 μM) or 1% BSA for 12 h, and then the CMs were collected to treat the spleen lymphocytes for 12 h. All the CD4^+^IFN-γ^+^ T cells or CD8^+^IFN-γ^+^ T cells were counted by flow cytometry analysis.

All data represent the means±s.e.ms. (**P<0.01, ***P<0.005; Student’s *t*-test).


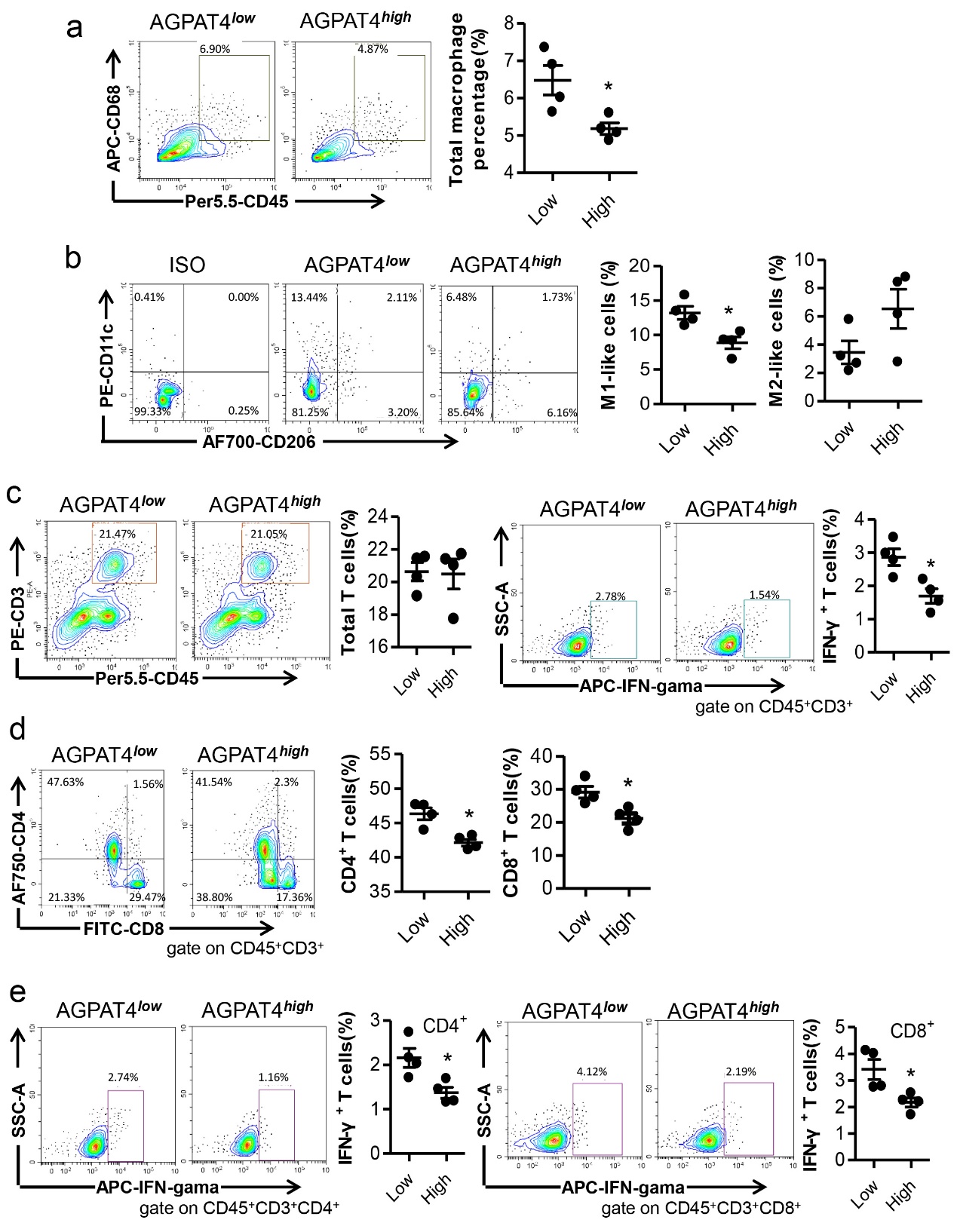


Figure. S6.

CRC tissues with lower expression of AGPAT4 have higher infiltration of M1-like macrophages and IFNγ-producing T cells

(**a-b**) Eight cases of fresh human CRC tissues were collected and subjected to IRS evaluation of AGPAT4 expression, according to which these samples were classified into two groups: AGPAT^low^ (n=4) and AGPAT^high^ (n=4). Total macrophages (**a**) and the subpopulation (M1-like and M2-like) (**b**) in each sample were determined by flow cytometry analysis.

(**c**) Total T cells and IFNγ^+^ T cells in the AGPAT^low^ and AGPAT^high^ CRC tissues described above in (**a-b**).

(**d**) Percentage of CD4^+^ and CD8^+^ T cells in the AGPAT^low^ and AGPAT^high^ CRC tissues described above in (**a-b**).

(**e**) Percentage of IFNγ^+^ cells in the CD4^+^ and CD8^+^ T cells in the AGPAT^low^ and AGPAT^high^ CRC tissues described above in (**a-b**).

Data in (**a-e**) shows the means±s.e.ms. (n=4, *P<0.05; Student’s *t*-test).


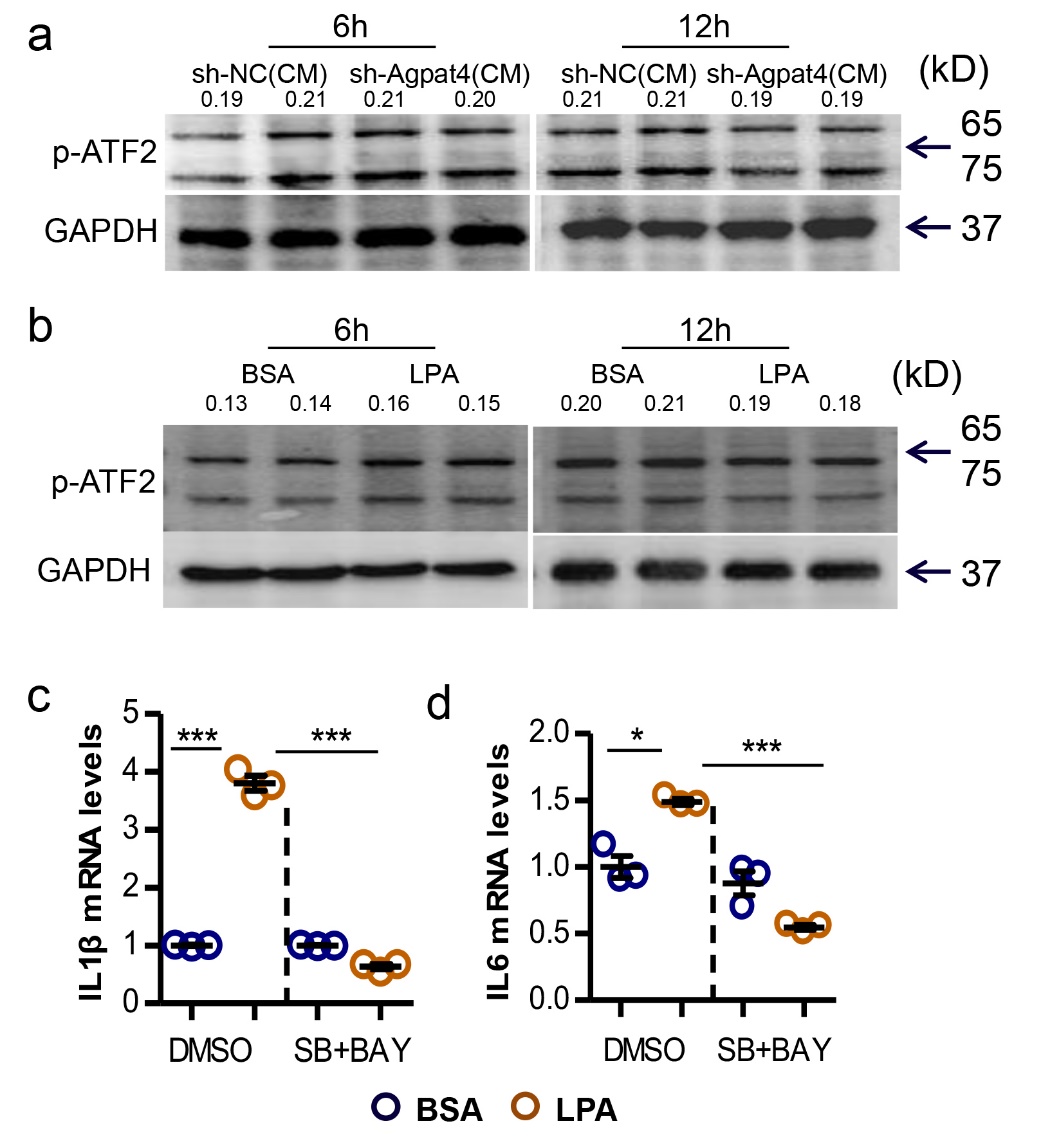


Figure. S7.

Agpat4 silencing in CRC does not affect ATF2 signaling in macrophages

(**a**) Immunoblotting assays of phosphorylated ATF2 (P-ATF2) in PMs treated with CMs from sh-Agpat4- or sh-NC-transfected MC-38 cells for 6 h (left) or 12 h (right).

(**b**) Immunoblotting assays of phosphorylated ATF2 (P-ATF2) in PMs treated with LPA (200 μM) or 1% BSA for 6 h (left) or 12 h (right).

(**c-d**) PMs were simultaneously pretreated with a NF-κB inhibitor BAY 11-7082 (10 μM) and a p38 inhibitor SB203580 (10 μM) for 4 h, and then additionally stimulated with LPA (200 μM) or 1% BSA for 12 h before harvested for Real-time PCR assays of IL-1β (**c**) and IL-6 (**d**) levels.

Data in (**c-d**) show the means±s.e.ms. (*P<0.05, ***P<0.005; Student’s *t*-test).


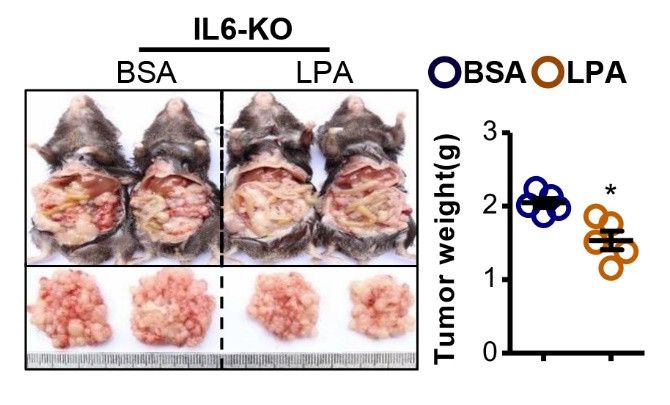


Figure. S8.

LPA suppresses CRC progression to some extent via IL-6

Six-week-old male IL6-KO mice were intraperitoneally engrafted with MC-38 cells (4×10^6^ cells in 100 μl PBS per mouse) on day 0, and then treated with LPA (50 mg/kg) or BSA as control on days 1, 3, 5, 7 and 9. Mice were sacrificed on day 15 and tumor nodules were observed. Data shows the means±s.e.ms. (n=5, *P<0.05; Student’s *t*-test).

Table S1.

Primer sequences for Real-time PCR

| Gene | species | Forward (5' → 3') | Reverse (5' → 3') |
| --- | --- | --- | --- |
| Gapdh | mouse | TGTGTCCGTCGTGGATCTGA | CCTGCTTCACCACCTTCTTGAT |
| Agpat4 | mouse | TCTTTCCAGTGTTGGCTGACT | GGGCTCAGGAAAGTGTCTCG |
| IL-1β | mouse | ACTCATTGTGGCTGTGGAGA | TTGTTCATCTCGGAGCCTGT |
| IL-6 | mouse | TCGTGGAAATGAGAAAAGAGTTG | AGTGCATCATCGTTGTTCATACA |
| IL-10 | mouse | GAGAAGCATGGCCCAGAAATC | CGCATCCTGAGGGTCTTCA |
| TNF-α | mouse | CTGAGGTCAATCTGCCCAAGTAC | CTTCACAGAGCAATGACTCCAAAG |
| Arg-1 | mouse | CTGAGCTTTGATGTCGACGG | TCCTCTGCTGTCTTCCCAAG |
| IFN-γ | mouse | CAGCAACAGCAAGGCGAAA | CTGGACCTGTGGGTTGTTGAC |
| Lpar1 | mouse | CATGGTGGCAATCTACGTCAA | AGGCCAATCCAGCGAAGAA |
| Lpar3 | mouse | CCACTTTCCCTTCTACTACCTGCT | GACGGTCAACGTTTTCGACACC |
| AGPAT1 | human | GGGGAGTTTGGGCTTTTCCTA | ACAAATCCATTCTGGCCACCT |
| AGPAT2 | human | TCAGGGAGAACGTGCCCATC | CACCTGCACTGTGACTGTTCC |
| AGPAT3 | human | CTCCAAGGTCCTCGCTAAGAAG | CCGCTTGCAGAACACAATCTC |
| AGPAT4 | human | CACGGAATGCACCATCTTCA | GAACCACGATGGCATTTTCCT |
| AGPAT5 | human | TGCGAAACAAGTTGCAGAGC | GCAGCAAATGCCTGACTAGC |
| GAPDH | human | CGGAGTCAACGGATTTGGTCGTAT | AGCCTTCTCCATGGTGGTGAAGAC |
